# Accurate prognosis for localized prostate cancer through coherent voting networks with multi-omic and clinical data

**DOI:** 10.1038/s41598-023-35023-9

**Published:** 2023-05-15

**Authors:** Marco Pellegrini

**Affiliations:** Institute of Informatics and Telematics (IIT), CNR, 56124 Pisa, Italy

**Keywords:** Predictive medicine, Urological cancer

## Abstract

Localized prostate cancer is a very heterogeneous disease, from both a clinical and a biological/biochemical point of view, which makes the task of producing stratifications of patients into risk classes remarkably challenging. In particular, it is important an early detection and discrimination of the indolent forms of the disease, from the aggressive ones, requiring post-surgery closer surveillance and timely treatment decisions. This work extends a recently developed supervised machine learning (ML) technique, called coherent voting networks (CVN) by incorporating a novel model-selection technique to counter the danger of model overfitting. For the challenging problem of discriminating between indolent and aggressive types of localized prostate cancer, accurate prognostic prediction of post-surgery progression-free survival with a granularity within a year is attained, improving accuracy with respect to the current state of the art. The development of novel ML techniques tailored to the problem of combining multi-omics and clinical prognostic biomarkers is a promising new line of attack for sharpening the capability to diversify and personalize cancer patient treatments. The proposed approach allows a finer post-surgery stratification of patients within the clinical high-risk category, with a potential impact on the surveillance regime and the timing of treatment decisions, complementing existing prognostic methods.

## Introduction

According to the World Cancer Research Fund International web site (https://www.wcrf.org/cancer-trends/prostate-cancer-statistics/), prostate cancer (PRC) is forecast as the second most commonly diagnosed cancer type in men (with 1.4 million cases worldwide) for the year 2022, and the 4th most commonly diagnosed cancer in the overall population (male and female). The ECIS—European Cancer Information System (https://ecis.jrc.ec.europa.eu) predicts an incidence of 363,000 new diagnosed PRC for the EU27 + EFTA area in the year 2025 and estimates a mortality of about 78,000 due to PRC (representing about 10% of the deaths due to cancer in the male population, ranking third as cause of death by cancer type in the EU27+EFTA male population). Siegel et al.^1^ report an estimate of 268,490 new cases of diagnosed prostate cancer for the year 2022 in the USA, and an estimate of 34,500 deaths due to prostate cancer (ranking second as cause of death by cancer type in the USA male population). About 15% of the localized prostate cancer diagnoses are clinically classified as high risk^2^ and thus require timely management decisions. This work concentrates on this fraction of the PRC patient population.

Prostate cancer is a very heterogeneous disease, from both a clinical and a biological/biochemical point of view, which makes the task of producing a stratification of patients into risk classes particularly challenging. In particular, it is important an early detection and discrimination of the indolent forms of the disease, from the aggressive ones, requiring closer surveillance^3, 4^. This report describes the application of a recently developed machine learning (ML) technique, called *coherent voting networks* to the problem of predicting an accurate prognosis for patients affected by prostate cancer (PRC).

Coherent voting networks (CVN) have been developed for the task of predicting the overall survival (OS) of breast cancer (BC) patients at 5 years after surgery, based on tissue transcriptomic fingerprints (mRNA), and depending on the specific adjuvant therapy adopted^5^. Since CVN is a general ML technique it is natural to extend such a technique to handling further cancer types (in this report, prostate cancer), further data type (including miRNA, CNA, microbiome, methylation, proteomics, etc.), different time points and different events of interest. Moreover, in this report, the CVN technique is further developed in order to provide additional theoretical grounding to some of the algorithmic phases involved. Specifically, the hyper-parameter optimization and feature selection phases (collectively indicated as model selection) are re-examined and a variation of the method by Andrew Ng^6^ to cope with model overfitting is shown to be both well grounded from a theoretical point of view and effective on PRC data. To the best of my knowledge, the application of Ng’s theory for prognostic purposes is new.

The improved CVN is used to develop multi-gene fingerprints for predicting the risk of Progress-free survival (PFS) of patients over several time points. The molecular data sets for the fingerprint discovery are provided by the TCGA consortium and consist of assays of prostate biopsies and tissue removed via radical prostatectomy in patients diagnosed with prostate adenocarcinoma, who had not received prior treatment for their disease (https://www.cancer.gov/about-nci/organization/ccg/research/structural-genomics/tcga).

Current diagnostic tests based on prostate-specific antigenes (PSA), Gleason score, Tumor stage, and other clinical measures often fail to distinguish between indolent and aggressive tumors, thus leading to over-diagnosis and over-treatment^7–10^. This adverse phenomenon has been the driving force behind much recent research aiming at integrating PSA with molecular profiling or finding new alternative prognostic features leading to a more accurate PRC prognosis.

As of 2021, Manjang et al.^11^ list at least 32 prognostic genic signatures for PRC, however only a handful have been thoroughly validated, and made into commercially available kits (including Oncotype Dx^12^, Prolaris^13^, Decipher^14^, Decipher PORTOS^15^, and ProMark^16^). Such commercial kits are increasingly included in clinical protocols and practice^17^.

This report contributes to the search for effective multi-gene prognostic fingerprinting by applying the CVN to several omic data sets from prostate cancer patients with the aim of learning a pool of effective fingerprints. Next, such fingerprints are applied to independent cohorts data sets to assess how performant these fingerprints can be (via a leave-one-out parameter optimization and bootstrapping performance evaluation). The reported results show remarkable promising performances in terms of Odds Ratio, Cohen’s kappa, and AUC, with good statistical significance, and a fine time resolution of 1 year. Moreover it is shown that standard clinical-pathological biomarkers can be combined with genomic biomarkers to improve predictive performance.

This paper is organized as follows. Section “Results” reports the main computational result on the performance of the proposed fingerprints. Section “Discussion” comments on the weak and strong points of the proposed methodology and places this work in the wider context of clinically useful prognostic tests for PRC. Section “Methods” recalls the main steps of the CVN construction and usage, and describes more in detail the novel model-selection techniques introduced in this report.

## Results

### Clinical features of the discovery population

The TCGA-PRAD dataset is used for training, validating, and testing the prognostic CVN in the discovery phase and determining the best performing multi-gene fingerprints. In Supplementary Table S9 it is reported the distributions of categorical attributes over the train, validate and test sets: progression-free survival status, tumor t-stage, tumor lymph node stage, radiation therapy, and reviewed Gleason sum.

In Supplementary Table S10 it is reported the distributions of numerical attributes: progression-free survival timing, age at diagnosis, tumor mutation burden index, duration of follow-up, and PSA level before surgery.

Overall, due to the randomized split of the patients, these features have similar distributions (mean, standard deviation) over the patient groups.

### Performance on TCGA-PRAD data

**Figure 1:**
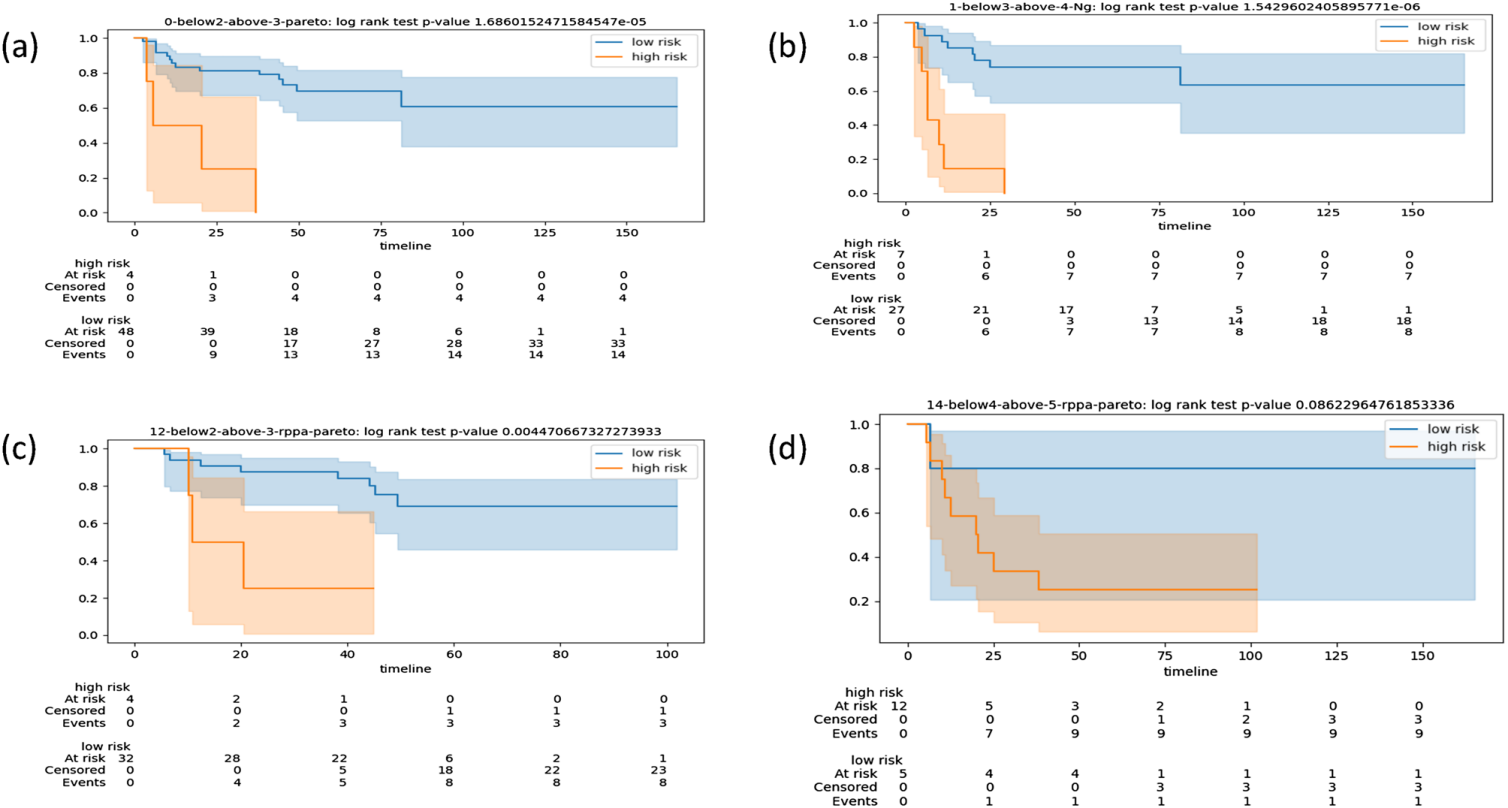
Kaplan-Meier plots for the stratifications of patients in the TCGA test cohort according to the optimal CVN obtained with different fingerprints. For each plot we note in the captions of the sub-figures: the label of the fingerprint, the type of data used in its derivation, the time frame (in years), where the first number refers to the threshold for high risk, the second number refers to the threshold for low-risk, and the model-selection procedure (Ng-based or Pareto-based). The timeline is in months. The acronym rppa stands for Reverse Phase Protein Array. (**a**) fp0, data mRNA, time frame 2–3, Pareto-based; (**b**) fp1, data mRNA, time frame 3–4, Ng-based; (**c**) fp12, data rppa, time frame 2–3, Pareto-based; (**d**) fp14, data rppa, time frame 4–5, Pareto-based.

**Figure 2:**
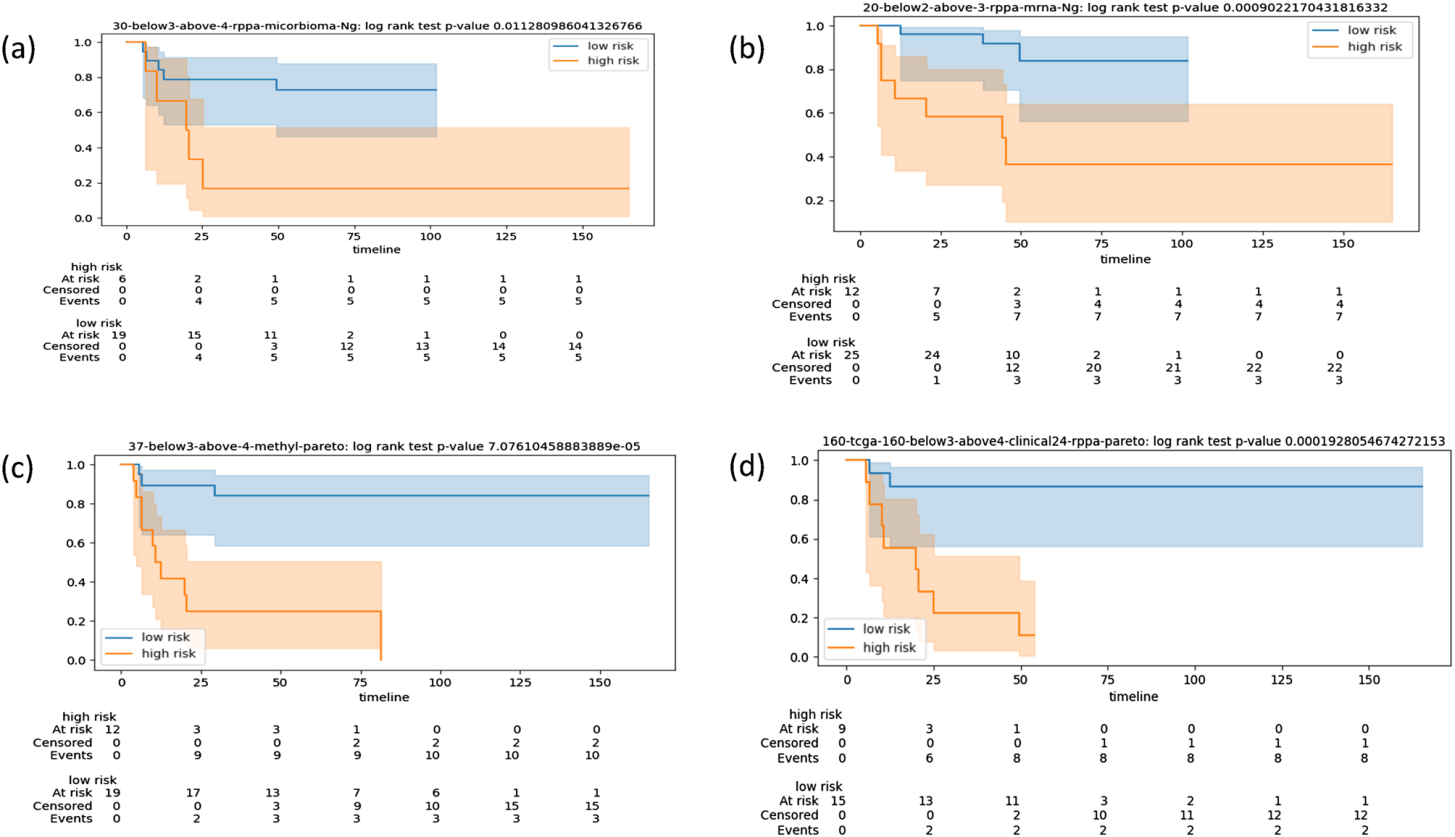
Kaplan-Meier plots for the stratifications of patients in the TCGA test cohort according to the optimal CVN obtained with different fingerprints. For each plot we note in the captions of the sub-figures: the label of the fingerprint, the type of data used in its derivation, the time frame (in years), where the first number refers to the threshold for high risk, the second number refers to the threshold for low-risk, and the model-selection procedure (Ng-based or Pareto-based). The timeline is in months. The acronym rppa stands for Reverse Phase Protein Array. (**a**) fp30, data rppa, time frame 3–4, Ng-based ; (**b**) fp20, data mRNA and rppa, time frame 2–3, Ng-based ; (**c**) fp37, data methylation, time frame 3–4, Pareto-based; (**d**) fp160, data mixed clinical and rppa, time frame 3–4, Pareto-based.

In Supplementary Table S2 seven fingerprints are reported giving the best performance for different input data types (mRNA, proteomics, and methylation) and different time frames (years defining thresholds for high-risk and low-risk patients: 2–3, 3–4, and 4–5. See “Methods”). For each of the seven fingerprints, the main measures of performance reported in Table 1 are odds ratio (OR), odds-ratio p-value and confidence intervals, Cohen’s kappa, AUC, AUC p-value and Confidence Interval, and the log-rank test p-value. The odds ratios range from a minimum of 12.0 to a maximum of 21.0, with an average 16.8, and all with significant p-values (except for fp14), geometric mean p-value 0.01. Cohen’s kappa ranges from a minimum of 0.29 to a maximum of 0.59, with an average 0.47. AUC ranges from a minimum of 0.62 to a maximum 0.79, with an average 0.72, with significant p-values (except for fp12) and geometric mean p-value 0.01. The log-rank p-values are all significant (except for fp14 which is borderline) and have a geometric mean p-value 0.0006. Fingerprint fp14 has a significant AUC p-value, while fp12 has significant OR p-value and log-rank p-value. Overall each fingerprint in Table 1 is statistically significant for at least one of the key measures. The Kaplan-Meier plots for these seven fingerprints on the TCGA Test dataset are in Figs. 1 and 2 giving a graphical display of the good separation properties of the selected fingerprints. Additional performance measures including PPV/NPV and Sensitivity/Specificity are reported in the GitHub project repository.

**Table 1:** Performance measures of seven fingerprints (Fp) on TCGA-PRAD discovery data. The performance is measured on the test data after training (on train data) and model selection (on validation data). We report the whether the fingerprint has bee selected via Pareto-based or Ng-based model selection. The table reports the fingerprint identifier (Fp), the reference year (year), the number of patients in the test set (n. pats), the number of no answers (n.a.), the odds ratio (OR), the OR 95% confidence interval, and its p-value, the Cohen’s kappa value, the area under the curve (AUC), its p-value (AUC-pval), the p-value of the log-rank test, and the lookahead number. For Ng-based model selection that does not use lookahead, the lookahead number is set to 0 by default. For averaging p-values the geometric mean is used , for other values the arithmetic mean. Results in this table are obtained with sw pipeline (a).

Fp	Year	n. pats	n.a.	OR	OR 95%CI	pval	Kappa	AUC	AUC pval	logrank pval	Lookahead
Fp_0_pareto	2–3	53	1	13	[1.2, 139.9]	0.03	0.29	0.72	0.005	1.60E−005	2
Fp_1_Ng	3–4	37	3	20	[2.0, 192.6]	0.01	0.54	0.7	0.01	1.50E−005	0
Fp_12_pareto	2–3	39	3	21	[1.7, 254.2]	0.01	0.47	0.62	0.16	0.004	1
Fp_14_pareto	4–5	19	2	12	[0.93, 153.8]	0.1	0.49	0.71	0.05	0.08	1
Fp_30_Ng	3–4	25	0	18.75	[1.6, 209.5]	0.01	0.53	0.72	0.03	0.01	0
Fp_20_Ng	2–3	39	2	17.14	[1.7, 172.0]	0.008	0.43	0.79	0.01	0.0009	0
Fp_37_pareto	3–4	31	0	16	[2.6, 96.4]	0.001	0.59	0.78	0.004	7.07E−005	1
Averages				16.8		0.01	0.47	0.72	0.01	0.0006

### Independent cohorts

In order to validate the selected fingerprints, their prognostic performance is measured on seven independent cohorts of PRC patients (listed in Supplementary Table S7) with a raw total of 744 patients. These independent data sets have been produced with several platforms and include as event endpoints: Overall survival (OS), Biochemical recurrence (BCR), Disease-free survival (DFS), or a category-based High-risk/Low-risk assessment. On these independent cohorts, the gene fingerprints are fixed and predictors are generated for leave-one-out (LOO) assays on the full range of hyperparameters for CVN, finally selecting the best performing configuration in terms of OR (or Cohen’s kappa), subject to a limit on the number of no answers below 15%. Since it is known that leave-one-out cross-validation has a low bias but a high variance in performance estimation of the generalization error, a bootstrap performance estimation of the selected configuration (fingerprint plus hyperparameters) is performed using the theory of Efron and Tibshirani^18^ (more details in the “Methods” section). Table 2 reports the combinations of data sets and fingerprints for which OR at least 8.0, and no answers below 20% of the number of patients in the bootstrapping assay is obtained. All results reported are statistically significant (below 0.05) in p-value for at least one key measure (OR or AUC). The Odds ratio ranges from a minimum of 8.33 to a maximum of 40.0 with average 17.09 and (geometric) mean p-value 0.003. Cohen’s kappa ranges from a minimum of 0.18 to a maximum of 0.65 with an average 0.4, while AUC values range from 0.61 to 0.88, with average 0.76 and (geometric) mean p-value 0.001. Interestingly, the best performance in terms of OR and kappa is attained for fp1 on data set GSE46602 which is the most balanced data set among the cohorts used in this study, having about 50% of high-risk and 50% low-risk patients. The selected fingerprint appears to have good prognostic performance across a wide choice of molecular measurement platforms, event end-points, and patients’ clinical conditions.

**Table 2:** Performance of the seven CVN selected fingerprints over seven independent cohorts of PRC patients using loo model selection and bootstrap performance evaluation. We report 21 combinations of cohort vs fingerprints attaining OR above 8.0. The table lists the independent cohort id (Dataset), the reference year (year) or the risk class for GSE37199, the fingerprint ID (Fp), the number of patients (n. pats), the average number of no answers (n.a.), the estimation of the Odds ratio (OR), the OR 95% confidence interval, its p-value (pval), and the estimation of Cohen’s kappa (kappa), based on the expected values of true/false positives and true/false negatives by the bootstrapping. The area under the curve (AUC) value and its p-value (AUC-pval) are measured for the consensus predictor obtained by the bootstrapping. GSE84042 is the only independent data set among those considered with methylation data fit for validating fp37. Proteomic fingerprints have been validated on mRNA data of independent cohorts. For averaging p-values the geometric mean is used, for other values the arithmetic mean. Results in this table are obtained with sw pipeline (b).

Dataset	year	Fp	n. pats	n.a.	OR	OR 95% CI	pval	kappa	AUC	AUC-pval
MSKCC	2-3	Fp_0	110	1.7	15.4	[4.05, 58.71]	0.0001	0.4	0.68	0.003
MSKCC	3-4	Fp_0	93	3.77	13.11	[2.71, 63.26]	0.0007	0.32	0.7	0.0006
GSE70769	2-3	Fp_0	39	10.1	12	[1.68, 85.70]	0.02	0.42	0.77	0.007
GSE46602	2-3	Fp_0	30	1.2	9.2	[1.38, 62.36]	0.06	0.33	0.72	0.01
GSE53922	2-3	Fp_0	89	14.32	8.33	[1.65, 41.90]	0.01	0.26	0.64	0.02
GSE46602	3-4	Fp_14	30	5.06	9	[1.50, 53.96]	0.03	0.43	0.78	0.005
GSE53922	3-4	Fp_14	90	26.08	20	[3.68, 108.53]	0.003	0.43	0.82	0.0001
MSKCC	2-3	Fp_1	110	3.9	8.66	[2.64, 28.40]	0.0007	0.37	0.69	9.70E-005
GSE46602	2-3	Fp_1	30	4.05	30	[4.22, 213.15]	0.002	0.59	0.86	0.0003
GSE46602	3-4	Fp_1	30	1.69	40	[4.48, 356.46]	0.0005	0.65	0.83	0.0009
GSE46602	2-3	Fp_12	30	3.7	25	[3.76, 166.22]	0.003	0.59	0.86	0.0006
GSE84042	2-3	Fp_12	65	7.3	18.33	[1.38, 242.77]	0.12	0.37	0.88	0.002
MSKCC	2-3	Fp_30	110	2.36	13.83	[1.82, 105.08]	0.02	0.18	0.61	0.04
GSE54460	3-4	Fp_30	31	1.47	20	[0.99, 401.64]	0.16	0.48	0.79	0.05
GSE46602	2-3	Fp_30	30	5.3	13.5	[2.21, 82.28]	0.01	0.47	0.82	0.002
GSE53922	2-3	Fp_30	89	7.66	12	[1.75, 82.08]	0.004	0.21	0.7	0.001
MSKCC	2-3	Fp_20	110	4.6	8.5	[2.43, 29.77]	0.001	0.34	0.67	0.006
MSKCC	3-4	Fp_20	93	2.2	22.47	[3.58, 140.98]	0.0006	0.32	0.7	0.001
GSE46602	3-4	Fp_20	30	0.5	30	[4.53, 198.56]	0.001	0.55	0.83	0.001
GSE53922	2-3	Fp_20	89	40	16	[3.16, 80.95]	0.002	0.43	0.79	0.0007
GSE37199	HR-LR	Fp_20	107	28.2	10.88	[2.26, 52.33]	0.001	0.33	0.69	0.001
GSE84042	4-5	Fp_37	145	20.4	19.9	[4.03, 98.86]	0.000017	0.46	0.87	1.90E-006
Averages					17.5		0.003	0.4	0.76	0.001

Of the seven independent cohorts used in this study, five are obtained via surgically removed tumor tissues (through either biopsies or radical prostatectomy), thus consistent with the specimens used in the discovery cohort. Two independent cohorts (GSE37199 and GSE53922) are based instead on blood samples of PRC patients. Unexpectedly, fingerprint Fp20 has significant discriminative power also on both of these test cases, however the number of no answers increases to 30–40% of the patient cohort.

Additional performance measures including confidence intervals, PPV/NPV, and Sensitivity/Specificity are in the GitHub project repository.

### Mixed clinical and genomic fingerprints

From the TCGA PRAD clinical data file 24 clinical/pathological features known to have prognostic power in prostate cancer were selected. These features were then appended to the omic molecular expression matrices and the fingerprint discovery pipeline was iterated. The fingerprint fp160 composed of three clinical parameters: Gleason primary score, tumor stage, psa, and two molecular protein expression levels for CDKN1B and NF2 has emerged as very concise and performant. Performance measures are reported in Table 3 both for the discovery pipeline and for the validation pipeline on independent cohorts. Figure 2d is the corresponding Kaplan-Meier plot. The AUC measure is 0.87 at p-value 0.001 on TCGA test data and is consistently confirmed in three independent cohorts bootstrap evaluations as well as in bootstrapping on the complete TCGA cohort. This mixed fingerprint attains better performance (in terms of AUC) with respect to the predictors obtained using the same discovery procedure starting from the 24 clinical/pathological features alone (data not shown), or from the genomic data alone (data in Table 1). The mixed fingerprint has also better performance than a fingerprint composed only of Gleason total score, tumor stage, psa, and age in terms of its stability in bootstrapping experiments (data not shown).

**Table 3:** Performance of the mixed clinical and molecular fingerprint fp160 on the TCGA PRAD (Reverse Phase Protein Array) rppa data set and on independent cohorts. In the notes, it is reported the software pipeline used, and whether the input data set has been equalized with a size ratio of the two labels up to 3-to-1 or 2-to-1. n.a. is the number of no answers. The time frame (years) is (3,4).

Dataset	fp type	n. pat	n.a.	OR	OR 95% CI	or pval	Kappa	AUC	AUC pval	Notes
TCGA-rppa	fp160	25	1	22.75	[2.6, 198.1]	0.002	0.64	0.87	0.001	sw (a), Pareto, lh = 1
TCGA-rppa	fp160	126	22	18.8	[6.9, 51.3]	2.1E-10	0.61	0.83	4.8E−9	sw (b)
GSE46602	fp160	30	0	28.8	[2.9, 284.7]	0.0007	0.6	0.85	0.0006	sw (b)
GSE70769	fp160	20	2	39.0	[1.8, 817.6]	0.01	0.67	0.88	0.01	sw (b), eq 3-1
GSE84042	fp160	36	6	22.0	[2.3, 204.7]	0.007	0.58	0.87	0.002	sw (b), eq 2-1

### Additional experimental results

A series of complementary tests and searches have been performed in order to support the novelty, relevance, and robustness of the proposed prognostic fingerprints and algorithms in the context of prostate cancer. In Supplementary Materials Section 1 and Supplementary Table S1, the genes in the selected fingerprints are analyzed for their functional associations with cancer in general (and prostate cancer in particular), finding that the selected biomarkers have often an experimentally demonstrated deep impact on cancer insurgence and progression. In Supplementary Materials Section 2 and Supplementary Table S3, it is shown that the performance of CVN is not achieved by a single state-of-the-art ML method, and that CVN has a consistent uniform good performance across a variety of data. In Supplementary Materials Section 3 and Supplementary Table S4, the analysis is restricted to patients classified as high risk or intermediate risk according to two established clinical staging systems, thus showing the capability of refining such classifications using CVN and the selected fingerprints. In Supplementary Materials Section 4 and Supplementary Table S5, it is explored the issue whether CVN would work as well when biomarker candidates are obtained by randomly probing the pool of differentially expressed genes, concluding that that the selected fingerprints do perform better than those obtained via a random process (for the fixed CVN algorithm). In Supplementary Materials Section 5 and Supplementary Table S6, the selected fingerprints are compared with several prognostic and predictive gene fingerprints in literature, finding minimal overlaps, thus confirming their novelty.

## Discussion

As research in prognostic predictions, in general, and for prostate cancer, in particular, is a vast subject with implications from several areas of biology and medicine, here comments on the relationship of this work with some issues arising in the relevant literature are given. Each issue is introduced by a short heading.

### Role of AI and ML in biomarker discovery

Alarcón-Zendejas et al.^19^ and Goldenberg et al.^20^ review recent advances in biomarker discovery for prostate cancer, indicating ML-based and AI-based approaches as opening a new dimension to research and opportunities for transferring new computational techniques in clinical practice in this area. This work pushes this view by extending the novel ML paradigm of the Coherent Voting Networks (CVN) with improved model selection techniques, and by applying it to the challenging problem of the prognosis of prostate cancer at a fine time granularity (year-to-year).

### Prognosis based on gene expression and proteomic data

This study uses mainly mRNA gene expression data sets obtained via high throughput assays as the primary source for prognostic biomarker discovery and validation. This technology is now mature and, over time, data on many cohorts of patients have become publicly available. The results on mRNA-based fingerprints appear to be robust w.r.t the specific technology used for measuring mRNA levels of expression. Interestingly, some of the best reported results are obtained from proteomic data obtained with Reverse-Phase Protein microArrays (rppa) assays^21^. Such proteomic data, although less abundant than mRNA expression data may have the advantage of representing a more accurate snapshot of the cell’s biological processes. This study has derived two fingerprints from mRNA data, three from proteomic data, one mixed with mRNA and proteomic data, and one from methylation data.

### Role of methylation in cancer

Many studies indicate that changes in DNA methylation contribute to cancer development and regulation. Cancers characteristically display extensive hypomethylation of DNA repeats as well as frequent focal DNA hypermethylation^22, 23^. Toth et al.^24^ attain good prognostic performance with a Random Forest algorithm, to discriminate patients according to eventual recurrence-free survival as an outcome, measured by PSA levels. However, the model they describe requires input from a large number of methylation sites (402 differentially methylated sites). The methylation-based fingerprint comprises just six methylation loci with performance validated in the independent GSE84042 methylation data set.

### MicroRNA, microbiome, and copy number alterations

MicroRNAs have been investigated as potential biomarkers for PRC prognosis as they can be derived also from liquid biopsies^25^, although the majority of studies still uses tissue-derived microRNA^26^. Experiments with microRNA data from the TCGA-PRAD cohort did produce fingerprints with statistically significant but suboptimal performance (data not shown) vs. those obtained via mRNA, rppa (Reverse Phase Protein Array), and methylation data. Similarly, statistically significant but suboptimal results were obtained with TCGA-PRAD CNA and microbiome data (data not shown). Smith and Sheltzer^27^ study the prognostic power of CNA in several cancer types, including prostate cancer, focusing on alterations of known driver genes. They used Cox proportional hazards analysis, concluding that very few mutations were significantly associated with patient outcomes. Their analyses suggested that, in general, cancer driver gene mutations lacked significant patient stratification power. The results in this study on the TCGA-PRAD CNA are consistent with these findings.

### Prognostic signatures through tissue classification

This study aims at predicting individual prognostic high-risk/low-risk stratification of patients along yearly time-frames in the first 5 years post-surgery/biopsy. Another form of prognostic study aims at a classification of the tumor tissues into sub-types, and then at using this information to derive broad prognostic indications. For example, Dhanasekaran et al.^28^ study the patterns of differentially expressed genes in normal adjacent prostate tissue (NAP), benign prostatic hyperlasia (BPH), localized prostate cancer, and metastatic, hormone-refractory prostate cancer, using unsupervised hierarchical clustering. Among the genes cited^28^ as strongly correlated with the above classification, two genes (MYC and CDH1) are also present in the selected fingerprints. Rhodes et al.^29^ produced a list of genes consistently up-regulated or down-regulated in several cohorts of prostate cancer patients with clinically localized prostate cancer versus benign prostate tissue. In this list, MYC is found but no other gene in the selected fingerprints. The inference is that, in all likelihood, the fingerprints in this study do not target the known PRC subtypes per se, but, instead, aim directly at the tracking the relevant biological process in tumor’s development (see also Supplementary Materials Section 1 and Supplementary Table S1).

### Prognosis based on clinical and histological data

Historically, histological and clinical parameters have been extensively studied in order to provide effective prognostic stratification of PRC patients. This line of research is now being supplemented with AI-based techniques. For example, Guinney et al.^30^ recently used crowdsourced challenges to improve prostate cancer prognostic models based on open clinical trial data, including 150 curated clinical variables, within the DREAM initiatives (Dialogue for Reverse Engineering Assessments and Methods). A hybrid approach is using genomic profiling to reduce the technical and subjective variability in the estimation of well-known clinical/histological parameters. For example, Wang et al.^31^ initially identify the candidate genes related to the Gleason score, then these genes are used to construct a LASSO Cox regression prognostic analysis model based on a 3 genes fingerprint (CDC45, ESPL1, and RAD54L). Predictors composed of mixed clinical and omic features were also considered, finding good and performance, confirmed in independent cohorts, for a fingerprint composed of three well known clinical parameters (PSA, Gleason primary score, and tumor stage) and expression levels for NF2 and CDKN1B. Interestingly these three clinical parameters were not pre-determined, but emerged from a pool of 24 clinical features. Moreover, the fact that these three clinical features are already routinely collected in practice, implies that just two additional ’omic’ expression measurements need to be collected (possibly by RT-PCR). Integration of clinical and genomic fingerprints has been shown to be beneficial also for the Decipher fingerprint^32^.

### Role of therapies

In the discovery cohort TCGA-PRAD, no patient received neo-adjuvant therapies prior to surgery/biopsy. About a quarter of the patients has a record of some treatment after surgery (radiation or pharmacological), which may have been administered after monitoring revealed the progress of the disease. Since the aim of this study is at predicting the duration of progression-free survival (PFS), and treatment data was not complete, no stratification of patients into treatment classes has been done. Moreover, note that this study is retrospective and the effect of personalized therapeutic choices can be detected more reliably within randomized clinical trials specifically designed for this objective.

### Multi-gene prognostic tests in clinical practice and guidelines

Beyer et al.^33^ recently compiled a systematic review of diagnostic and prognostic biomarkers in prostate cancer, with emphasis on those likely to progress towards clinical practice. The proposed multi-gene biomarker fingerprints may be useful within the prostate cancer management work-flow as a PRC risk stratification decision point, following a prostate biopsy/surgery, thus it can be hypothesized a potential future use akin to that of the current kits such as Promark, Oncotype Dx, Prolaris, and Decipher.

### Multi-omic signatures

Fraser et al.^34^ study in-depth the class of localized, non-indolent prostate cancer and propose a multi-modal pool of biomarkers to predict disease relapse as indicated by BCR (this signature includes clinical, gene expression, methylation sites, SNV, and CNA). Interestingly, their method was effective in predicting eventual relapse with AUC 0.83 (See Fig. 10(h)^34^). However, when it was applied to detect early relapse (relapse by month 18) it did not perform well (log-rank test p = 0.14) (See Fig. 10(g)^34^). In contrast, the proposed signatures are effective within the first 2–5 years since surgery/biopsy, with 1-year resolution. Most of the proposed fingerprints are composed of one molecular type, except fp20 which is composed of two, and fp160 composed of clinical and genomic (Reverse Phase Protein Array) markers. Several recent studies have focused their attention on providing refined risk stratifications in the early years after primary treatment. Fu et al.^35^ propose an 18-genes genomic fingerprint for prediction of recurrence with AUC performance values of 0.747, 0.827, and 0.851 respectively after 1-, 3-, and 5-years from surgery in the GSE46602 independent cohort. Zhou et al.^36^ report prognostic accuracies for 3- and 5-year BCR-free survival of AUC 0.68 and 0.713, respectively, for a 26-patient independent cohort. Results reported in Tables 2 and 3 show that some of the fingerprints reported in this study may attain higher AUC values with shorter fingerprints.

### Tumor tissue vs liquid biopsies

Blood samples have several advantages with respect to tumor tissue samples as biospecimen of choice for prognostic purposes, and several blood-based prognostic signatures have been proposed for prostate cancer^37, 38^. In particular issues relative to PRC multiclonality and inter-tumor heterogeneity may limit the use of tissue biopsies as a source of reliable prognostic tests^39^. These issues may be mitigated in blood samples. Testing the selected fingerprints on independent cohorts with data from blood samples (GSEGSE53922 and GSE3719), it was found that one fingerprint (fp20) retains prognostic power also in both of these cohorts, although with a higher percentage of no answers. As the biological and transcriptional interplay of primary prostate adenocarcinoma with eventual bone metastasis affecting several components of blood is complex and not well-understood^37, 38^, I expect that better results may be obtained by using blood samples (and/or its components, e.g. extracellular vescicles, serum, PBMC, and CTC) directly as the target for the biomarkers discovery phase.

### Castration-resistant prostate cancer

One independent cohort (GSE53922) is composed mainly of patients at the stage of Castration-Resistant Prostate Cancer (CRPC). It was found that fingerprints fp14, fp20, and fp30 are prognostic with good performance also for this sub-class of PRC patients, although, in this case, further data is likely needed to confirm this finding. For fp20 also partial support comes from the result on cohort GSE37199 where fp20 can discriminate CRPC from the indolent form of local PRC.

### Role of the pool of selected genes in cancer progression

Many of the genes in the eight fingerprints been studied individually for their role in cancer (of any type), and they affect functionally important cancer biological processes, as determined via knock-out experiments in cell lines and/or animal models of cancer. In some cases, their gene expression is directly modulated by a microRNA with an important role in cancer progression. Although this is not yet sufficient to establish causal relationships between the expression of these genes and tumor development, it is a good stepping stone towards a more complex type of analysis that integrates bio-networks and causality relationships more explicitly in the model.

### Limitations of the current CVN approach

The main limitation in the current state of the CVN methodology is that the biomarker discovery phase is based on the trisection of the discovery cohort into training, validation, and testing sets (roughly half, one quarter, and one quarter, respectively), while the performance of the selected model can be measured reliably only on the testing set. Thus the size of the discovery cohort needs to be rather large in order for the testing set to be sufficient to attain statistical significance. It is an open line of research to extend the model-selection phase to reach statistical robustness with fewer initial samples.

### Comparative evaluation of fingerprints

One natural question is whether, among methylation, mRNA, proteomic, and mixed fingerprints, one data type outperforms the others in the context of prostate cancer prognosis. The answer to this question is mostly dependent on trade-offs across different concerns. For example, data from Table 1 and Supplementary Table S2 on the discovery cohort TCGA-PRAD indicates that fingerprints based on data from proteomic assays (rppa) might have a greater dynamic range, covering predictions of PFS for all time frames from year 2 to year 5, while the methylation-based fingerprint fp37 is performant in only one specific time frame. Thus, in case the dynamic range of the prediction is considered a key feature of a prospective clinical test, rppa data might be the optimal choice, with an assay aiming at measuring several rppa-based fingerprints at once. A second concern is the practicality of handling the bio-specimens and extracting the molecular species to be analyzed. Here the advantage of adopting a mixed mRNA and rppa fingerprint fp20, attaining AUC value 0.79, which is marginally higher than AUC values for the pure mRNA or rppa fingerprints on TCGA-PRAD data, should be weighed against the disadvantage of handling pipelines for two parallel molecular assays. The mixed clinical and rppa-based fingerprint fp160 has a special status since the three clinical parameters (Gleason primary score, tumor stage, psa) are routinely collected in the current clinical protocols, thus the marginal cost of setting up an assay for fp160 is associated with measuring the levels of CDKN1B and NF2. The performance levels for fp160 measured in AUC are very high and consistent across the discovery and independent cohorts.

## Methods

### Overview

Supplementary Fig. S2 shows a schematic depiction of the two main software pipelines used to derive the results reported in this work. In this section, it is given a summary of the main principles of the Coherent Voting Network paradigm while more algorithmic details are in Pellegrini^5^. Novel algorithmic features described below include a model selection module based on a theory by Andrew Ng for avoiding model overfitting, and the implementation of a bootstrapping module in a train-test setting according to the work by Efron and Tibshirani^18^.

### Discovery cohort and independent validation cohorts

The discovery cohort is the TCGA-PRAD (2018) data set downloaded from cbioportal (https://www.cbioportal.org) (additional clinical data has been obtained from UCSC Xena repository (https://xena.ucsc.edu)). The procedures for sample selection and processing are described in detail in the paper by Abeshouse et al.^3^ and its Supplementary files. Briefly, surgical resection biospecimens were collected from patients at the participating institutions diagnosed with prostate adenocarcinoma, who had not received prior treatment for their disease (chemotherapy, radiotherapy, or hormonal ablation therapy). The specimens comprise primary tumor tissue, normal solid tissue, and blood-derived normal. Pathology quality control was performed on each tumor and normal tissue specimen from a frozen section slide. Hematoxylin and eosin (H &E) stained sections from each sample were subjected to independent pathology review to confirm that the tumor specimen was histologically consistent with the allowable prostate adenocarcinoma subtypes and the adjacent normal specimen contained no tumor cells. Computational pipelines include batch effect analysis and correction. Note that this study uses only the primary tumor-tissue data and clinical data. Some technical details of the data acquisition technologies are summarized in Supplementary Table S7. Although TCGA data was not originally collected for survival analysis, ex-post quality control studies by Liu et al.^40^ show that TCGA PRAD data for PFS is of high quality and can be safely used for prognostic purposes. A synopsis of independent cohort’s patient features is in the “Supplementary Materials” (Sections 6 and 7).

Extending the methodology in Pellegrini^5^, each patient is annotated with a risk class, taking censoring into consideration, setting progression-free survival below 12*t* months (year *t* and below) as high-risk, and progression-free survival above $12(t+1)$ months (year $t+1$ and above) as low-risk. For convenience, in this study *t* takes consecutive integer values 2, 3, 4 and 5; and each specific time frame is denoted with the pair $(t , t+1)$.

### Coherent voting networks

The Coherent Voting Network (CVN) is a supervised learning algorithm introduced by Pellegrini^5^ and applied to the classification of breast cancer patients into prognostic survival categories (low risk/high risk of overall survival above/below 5 years) after surgical removal of the tumor^5^. The Coherent Voting Network is designed explicitly to uncover non-linear, combinatorial patterns in complex data, within a statistically robust framework. Moreover, the *coherent voting communities* mechanism can be seen as a ’post hoc’ result explanation approach, providing a certificate justifying the survival prediction for an individual patient, thus facilitating its acceptability in practice, in the vein of explainable Artificial Intelligence (See discussion in “Supplementary Materials”).

In a nutshell, CVN can be seen as a generalization of the notion of guilt by association (GbA) in biological networks, where an unlabeled patient node receives a predicted label by collecting the vote of many dense communities of labeled patients and genes to which the unlabeled patient node belongs. The CVN algorithm also seeks a minimal number of genes with the property of allowing a coherent vote of high accuracy on the labeled nodes, and thus such a minimal set represents arguably a good candidate fingerprint to be performing well also on predictions for the unlabeled nodes. A schematic depiction of the workflow for the main CVN algorithm is in Supplementary Fig. S1. Further details can be found in Ref.^5^ [Supplementary Materials].

As in many complex ML paradigms, the CVN depends on a number of inner parameters, and thus it is important to do properly both feature selection (i.e. the selection of the fingerprint genes) and hyper-parameter optimization. These two tasks are called together the *model-selection* phase.

The input cohort of patients is split randomly into a training set, a validation set, and a test set (of size roughly 1/2, 1/4 and 1/4). Then the algorithm proceeds in three phases. In Phase I the CVN is applied to the training set (with full knowledge of the training patient survival labeling) in order to produce a list of candidate gene fingerprints (typically a number between 30 and 60 candidates in this paper). In phase II, the candidate fingerprints, the training set, and the validation set (with partial knowledge of the patient survival labeling for the validating set) are used together to do model-selection and fix both the fingerprint and the hyper-parameter configuration that minimizes the generalization error (or other performance target measures). Finally, in Phase III the single selected CVN is applied to the test set to measure the effective generalization error. The test set is a set of patients not used in phases I and II, thus unlikely to suffer from overfitting.

Pellegrini^5^ noticed that the standard model selection method suffers from a particular type of overfitting discovered by Ng^6^ as an effect of having a large number of hypotheses to choose from. This issue was solved by introducing a Pareto stratification^5^ of the models, and by using the notion of a limited and controlled lookup of test data during the model-selection phase (phase II). The lookahead number 1 corresponds to the standard model selection, while it was considered acceptable also lookahead numbers less or equal to 4, thus overfitting is prevented by using a controlled information leak.

The fingerprints so selected were next further validated in independent cohorts of cancer patients, thus showing that the Pareto-based model selection did perform well empirically.

The main technical contribution of this paper is a new look at the problem of model selection by generalizing and expanding the approach proposed by Ng^6^, as described in the next section. In practice, both the Pareto-based model selection and the Ng-based model selection are used to attain the results shown in this paper.

Missing data and censored patients are handled as described in detail in Pellegrini^5^.

### Ng-based model selection

In Ng^6^ it is described the following phenomenon. One has many predictive models (hypotheses) to choose from and uses cross-validation on a pool of validation data in order to select the hypothesis minimizing the cross-validation error, as a representing a hypothesis hopefully minimizing also the generalization error (to be evaluated on a different independent testing set drawn from the same distribution). Ng shows that when the number of hypotheses to choose from is large a form of over-fitting occurs so that the hypothesis minimizing the cross-validation error is a poor predictor of the generalization error. Next, an algorithm called LOOCVCV is proposed to cope with this phenomenon^6^. LOOCVCV is based on estimating the number $\hat{n}$ so that choosing the hypothesis with the smallest cross-validation error in a random subset $H'$ of size $\hat{n}$ of the initial set *H* of hypotheses has the minimum expected misclassification error. Having the estimate of $\hat{n}$, this value is then used in an index-scaling approach to select one of the hypotheses in a ranked list (by cross-validation error) of the initial *H* hypotheses.

The LOOCVCV method is modified and generalized in four aspects.

1. Optimization of the expected generalization value of functions different from the generalization error, in particular Cohen’s kappa measure (and variations of it).
2. Simplification of the handling of ties in the ranking of *H* by using lexicographic sorting of the value of a function paired with the index of the hypothesis.
3. Skipping the index-scaling approach to the hypothesis selection by recording in the computation process of the estimate of $\hat{n}$, the hypothesis having the largest (smallest) contribution/effect when the aim is at maximizing (minimizing) a target function.
4. probabilities of events are computed exactly via binomial coefficients, not in a quick but approximate fashion^6^.

The presence of possible no-predictions introduces some complications, as the Cohen kappa can be changed in several different ways. Four versions of the kappa function differing in the way they handle the no answers are computed. The first solution is to apply the standard Cohen’s kappa functional just ignoring the no answers. The second solution is to scale the first solution by the fraction of predictions. The third solution is to apply Gwet’s version of kappa^41^. Finally, a mixed version is considered that uses the second function for a number of no answers below 15% and the third version when the number of no answers is above 15%. These four measures are all in the range $[-1, +1]$. In order to select dynamically one of the four measures, each of them is normalized with respect to its own empirical distribution via a z-score. Among these four functions the function realizing the largest z-score (i.e. scaled displacement from the respective mean) is chosen.

### Pareto-based model selection

In Ref.^5^ [Suppl. Materials, page 25] the *Pareto-based* model selection process is described in detail. Here we give a summary to compare it with the Ng-based selection procedure. Each configuration of CVN on the Validating set is mapped to a point in 3D space representing its performance profile (number of hits, quality score, fraction of answers), where the quality score is either Cohen’s kappa or the Odds Ratio. Duplicated points are removed. For this set of points, the optimal (maximal) Pareto front is computed, and then the computation is iterated on the residual set. This process produces a Pareto stratification of the points. Within each stratum, the points are sorted by the quality score. This produces a total ordering of the points. Next, using this order, we compare the quality score obtained on the Validation set and the Test set for corresponding configurations. This comparison stops when either the Test quality score is better than the Validation quality score, or it is within a relative displacement of 0.2. The number of such comparisons is the *lookahead number (lh)* and it measures the controlled information leakage we allow to balance the performances of the validation and test sets. Not that lh $=1$ corresponds to the classical selection without information leakage. Low *lh* numbers $\le 2$ have been found for the fingerprints in Table 1.

### Bootstrapping

The independent cohorts used to validate the chosen fingerprints are smaller than the TCGA-PRAD cohort used to discover them. Therefore splitting these data into three sets risks producing results lacking statistical significance just due to the small numbers involved. For this reason, a different common machine learning paradigm is applied: the leave-one-out (LOO) approach to hyper-parameter optimization (now the features—genes—are fixed), and bootstrapping to evaluate the quality of the chosen configuration^42^.

Bootstrapping is a very general technique with deep theoretical support and extensive practical applications. In the context of cross-validation, the formalism by Efron and Tibshirani^18^ can be adopted. In particular, notice that the formula for the leave-one-out bootstrap error estimation (which is the smoothed version of the standard cross-validation estimation of the prediction error) can be applied to obtain smoothed estimates of any function that is a sum (linear combination) of the single error indicator functions for the elements of the testing set. Therefore bootstrap estimates of the relevant quantities: TP (True Positive), FP (False Positive), TN (True Negative), FN (False Negative), and NA (No Answers) can be made with this approach. From these values, estimates of the bootstrapped odds ratio and kappa are computed. Note that the area under the curve (AUC) does not have the required functional form for the application of the theory^18^. All the prediction maps produced in the bootstrap process are collected and for each patient in the input set a consensus prediction is produced that is the majority of the predictions in the collections of bootstrap maps. Finally, the AUC of the consensus prediction map is computed using the equivalence to the Wilcoxon-Mann-Whitney U-Statistic.

In standard bootstrapping the sampling in a set of *n* items is done by sampling uniformly at random *with replacement* $m=n$ times. Most of the bootstrap theories would carry on using a number of samples $m \ne n$ (see e.g. Bickel et al.^43^ for the correction to the theories need in this case). Note that the only practical effect of sampling in the context of this study is to partition the input set into an in-set and an out-set For the bootstrapping experiments, the value $m=3n$ is set, which ensures sufficient variability in the size of the out-sets (used for testing) while ensuring that the in-sets (used for training) are sufficiently stable. For the mixed clinical and genomic fingerprint on TCGA data, the value $m=1.38n$ is used ensuring that the expected size of the test subset of patients is 1/4 of the total in each bootstrap round, thus with a split close to the initial train-validate-test setting. The results in Table 2 are obtained for $B=200$ and are stable with respect to the number *B* of bootstrap iterations.

### Ethics approval and consent to participate

Patients were not directly involved in the study. All data used in this study is in the public domain and was obtained with the appropriate consent.

## Conclusions

This report has two main contributions. From the methodological point of view, the CVN (Coherent Voting Network) paradigm is extended by providing a novel robust model selection technique to overcome the danger of overfitting, inspired by a method of Andrew Ng. Next, the improved CVN methodology is applied to tackle the problem of stratifying prostate cancer patients in risk classes (for adverse events within 2–5 years from surgery/biopsy). Several candidate genomic fingerprints are produced to cover different time-frames at a 1-year resolution using of different omic data (mRNA, Reverse Phase Protein Array, and methylation) and clinical data. These multi-gene fingerprints can help in deciding the monitoring regime to be applied to prostate cancer patients, within an established clinical decision process. Many of the biomarkers in the proposed pool of genes are known individually as cancer hallmark genes or they are shown functionally involved in cancer using animal models or cell lines. The proposed fingerprints appear to be robust in tests with several independent cohorts. However, the task of measuring the proposed biomarkers in an accurate, reproducible, and cost-effective way for a clinical setting (e.g. via RT-PCR) is left as future research.

## Supplementary Information

Supplementary Information.

## Data Availability

Data supporting the findings of this study are available from the GitHub repository https://github.com/MarcoPellegriniCNR/Coherent-Voting-Network-for-PRC-prognosis.

## Code availability

Custom software and code availability is to be agreed via licensing contracts with the National Research Council of Italy.
