# Supplementary material for: Accurate prognosis for localized prostate cancer through coherent voting networks with multi-omic and clinical data
Source: Sci Rep. 2023 May 15;13:7875. doi: 10.1038/s41598-023-35023-9 (PMC10185505; doi:10.1038/s41598-023-35023-9)
Supplement: Supplementary file 1 — Supplementary Information. [file 41598_2023_35023_MOESM1_ESM.pdf]

Accurate prognosis for localized prostate cancer through coherent voting networks with multi-omic and clinical data – Supplementary Information

Marco Pellegrini[1,*]

[1]Institute of Informatics and Telematics (IIT), CNR, Pisa, 56124, Italy
[*]email: marco.pellegrini@iit.cnr.it, ORCID: 0000-0003-3151-9481

ABSTRACT

Supplementary text and tables for: Accurate prognosis for localized prostate cancer through coherent voting networks with multi-omic and clinical data.

Contents

1 Biological relevance of the genes in the fingerprints **1**
1.1 Human Protein Atlas and COSMIC 1
1.2 Fingerprint Fp0 2
1.3 Fingerprint Fp1 2
1.4 Fingerprint Fp12 2
1.5 Fingerprint Fp14 3
1.6 Fingerprint Fp30 3
1.7 Fingerprint Fp20 3
1.8 Fingerprint Fp37 3
2 Comparisons with Auto-weka predictors **3**
3 Performance on clinical stratifications **4**
4 Random fingerprints **4**
5 Overlap with published multi-gene signatures **4**
6 Independent validation cohorts: tumor-based samples **4**
7 Independent validation cohorts: blood-based samples **5**
8 CVN as an explanation tool **6**
9 Comparing the Pareto-based and the Ng-based model selection **6**
References **6**

1 Biological relevance of the genes in the fingerprints

In this section, it is reported the outcome of investigating the role of each gene of the selected multi-gene fingerprints in the progress of prostate cancer. I could not find evidence that significant subsets of the genes in the selected fingerprints have been analyzed together previously in the context of prostate cancer. First, two established online databases are used to explore the prognostic power and the oncological annotations of each gene separately with respect to cancer in general. Next, a literature search is done for articles reporting on direct or indirect functional associations of each gene to prostate cancer (or to solid tumors, more widely).

1.1 Human Protein Atlas and COSMIC

Searches of the protein-coding genes in The Human Protein Atlas database [https://www.proteinatlas.org] show evidence of prognostic power (relative to eventual OS) for 29 genes out of 37 in some cancer types (mostly kidney) (see Supplementary Table S1). This database records only one gene as having prognostic power in prostate cancer (for eventual OS). Notice however

that a recent study[1] on the TCGA data quality for survival analysis indicates that the TCGA-PRAD Overalla Survival (OS) annotations may be deficient, due to relatively short follow-up, thus the lack of prognostic power for OS for each individual gene in prostate cancer may be explained. TCGA-PRAD records for PFS are of good quality, in contrast.

The COSMIC (Catalogue Of Somatic Mutations In Cancer) database [https://cancer.sanger.ac.uk/cosmic/] was searched for annotations (see Supplementary Table S1), and seven fingerprint genes annotated as cancer "hallmark genes" were found. Nine genes were annotated as "mouse genes" [Mouse genes are listed in the Candidate Cancer Gene Database[2] recording functional effects in cancer mutagenesis experiments supporting the designation of the gene as causative in cancer.]. Two genes were annotated as "census genes" [Census genes possess a documented activity relevant to cancer, along with evidence of mutations in cancer that change the activity of the gene product in a way that promotes oncogenic transformation.]. The hallmarks are concentrated in fp12 and fp30, which share many genes, and fp14. Mouse and census genes are abundant in fp0 and fp1.

1.2 Fingerprint Fp0

Fingerprint fp0 consists of six genes, namely: CHST1, GHRL, MAK, RAB11FIP4, RPEL1, and ZEB1. Of these, four (GHRL, MAK, ZEB1, and Rab11-FIP4) have been studied in cell and animal models of PRC, one (CHST1) has been included in a published fingerprint[3], while one (RPEL1) does not appear to have been a focus of study in relation to PRC.

Ye et al.[4] study PC3 cell lines and *in vivo* mouse models of PRC showing that GHRL mRNA gene expressions and protein levels are increased in invasive PRC. Live imaging in mice models showed that there were different signal intensities of GHRL/GHSR peptide binding in tumor areas with different invasiveness.

Wang et al.[5] report that MAK dual phosphorylation of the conserved TDY motif is required for MAK kinase activation and that this phosphorylation displays a dynamic pattern during the cell cycle. MAK also acts as a negative regulatory kinase of APC/C^{CDH1}. Interestingly, the CDH1 gene also emerges in one of the selected prognostic fingerprints.

Orellana et al.[6] report that ZEB1 expression correlates with Gleason score in PRC samples and that expression of ZEB1 regulates epithelial–mesenchymal transition and malignant characteristics in PRC cell lines.

He et al.[7] knocked-out Rab11-FIP4 in PANC-1 pancreatic cancer cells using the CRISPR/Cas9 system and found that this alteration inhibited cell growth, invasion, and metastasis, and arrested cell cycle progression, but did not alter apoptosis.

1.3 Fingerprint Fp1

Fingerprint fp1 has 7 genes, namely: ASH1L-AS1, C1orf88, DBN1, HRSP12, MAFG, SNORA18, and TRIM65. Two of these have been studied for their role in cancer development. Ye et al.[8] performed rescue assays on PRC cell lines showing that MAFG may play a key role in facilitating PRC progression. Wang et al.[9] performed knockdown of TRIM65 in two lung cancer cell lines, SPC-A-1 and NCI-H358, resulting in a significant reduction in cell proliferation, migration, invasion, and adhesion with an increase in G0-G1 phase arrest and apoptosis.

1.4 Fingerprint Fp12

Fingerprint fp12 has seven genes/proteins, namely: CDKN1B, MAPK9, MYC, NDRG1, NF2, RB1, and SCD. All of them are known to be involved in PRC progression from cell lines and *in vivo* animal models.

Sirma et al.[10] use large tissue microarray (TMA) from 4699 hormone naive prostate cancers, obtained from patients who had undergone radical prostatectomy, and showed that the loss of CDKN1B/p27 expression was correlated with ERG fusion-negative tumors. The authors however could not identify a direct effect of p27 expression on prostate cancer phenotype or patient prognosis.

Xu et al.[11] review the role of the JNK family (including JNK1, JNK2 (alias MAPK9), and JNK3) in prostate cancer progression The JNK family has been shown to activate multiple substrates to modulate apoptosis, proliferation, tumorigenesis, and inflammation in response to various stimuli, with emerging evidence indicating the significant roles of the JNK family and androgen receptor in prostate cancer development.

Koh et al.[12] review a series of recent studies indicating that MYC appears to be activated at the earliest phases of prostate cancer (e.g., in tumor-initiating cells) in prostatic intraepithelial neoplasia, a key precursor lesion to invasive prostatic adenocarcinoma. This phenomenon is evident also in genetically engineered mouse models.

Sharma et al.[13] report experimental and clinical evidence suggesting that N-myc downregulated gene 1 (NDRG1) functions as a suppressor of prostate cancer metastasis. Their conclusions are based on a three-dimensional invasion assay and an *in vivo* metastasis assay for human prostate xenografts.

Several studies show that the inactivation of NF2 contributes to the progression of cancer toward a highly invasive and chemoresistant state[14].

Han et al.[15] engineered RB1-depleted C4-2 cell and showed that RB1 silencing resulted in significantly increased cell proliferation and decreased growth response to enzalutamide, a potent AR antagonist.

Fritz et al.[16] show that pharmacological inhibition of SCD1 activity limits lipid synthesis and results in decreased proliferation of both androgen-sensitive and androgen-resistant PC cells.

1.5 Fingerprint Fp14

Fingerprint fp14 had 6 genes/proteins, namely: CDH1, DIABLO, EGFR, GAB2, PRKCA, and RPS6KB1. For all of them, there is evidence of their involvement in key cancer development processes.

E-Cadherin (CDH1) is linked with low-penetrance susceptibility that is important in the development of cancer[17]

Kim et al.[18] demonstrate that the interaction between Smac/DIABLO and Survivin in the nucleus is an important step for suppressing the anti-apoptotic function of Survivin in Docetaxel-induced apoptosis for DU145 prostate cancer cells.

Nastaly et al.[19] indicate EGFR is a stable, EMT-independent, marker of PRC metastasis to rigid organs, in particular bones.

Tanaka et al.[20] report that activation of protein kinase C (PKC) by phorbol esters or diacylglycerol mimetics induces apoptosis in androgen-dependent prostate cancer cells.

Hussein et al.[21] report that suppression of ribosomal protein RPS6KB1 by Nexrutine increases the sensitivity of prostate tumors to radiation both in vitro in multiple PRC cell lines and in the Transgenic adenocarcinoma of mouse prostate model (TRAMP).

Quiao et al[22] use gene chip technology to screen differentially expressed genes in PC-3 human prostate cancer cells following GRB-associated binding protein 2 (GAB2) gene knockdown, and show that GAB2 regulates several key pathways for PRC insurgence and development.

1.6 Fingerprint Fp30

Fingerprint fp30 consists of six genes/proteins, namely: CDK1, CDKN1B, CLDN7, MYC, NF2, and SCD. All of them are involved in prostate tumor development. Note that many genes of fp30 are shared with Fp12. Two genes specific of this fingerprint are *CDK*1 and *CLDN*7.

Chen et al.[23] report that increased CDK1 activity is a mechanism for increasing both Androgen Receptor expression and stability in response to low androgen levels in androgen-independent PCas.

Zheng et al.[24] show that CLDN7 can regulate the expression of a tissue-specific protein, the prostate-specific antigen (PSA), in the LNCaP prostate cancer cell line

1.7 Fingerprint Fp20

Fingerprint fp20 consists of BAK1, PTCHD4, FANCC, FBRSL1, OMP, SULT1C3, and CDKN1B. Two genes in fingerprint fp20 are known to have functional associations with PRC development: BAK1 and CDKN1B.

Shi et al.[25] showed that transfection of synthetic miR-125b stimulates androgen-independent growth of CaP cells and down-regulates the expression of BAK1.

1.8 Fingerprint Fp37

Fingerprint fp37 consists of six methylation loci (listed in Supplementary Table S2). Using Illumina HumanMethylation450 BeadChip annotations the genes most likely affected by the methylation sites in fingerprint fp37 were identified.

The methylation site cg02928644 is annotated in the database http://www.ewascatalog.org (The MRC-IEU catalog of epigenome-wide association studies) as linked to sex and age, but lacks association with a protein-coding gene.

The other five methylation sites of fp37 are associated with the genes CCR10, NRN1, NPR3, C14orf23, and ATXN7L1. Some of these genes have been studied in relation to other types of tumors, however, their role in prostate cancer is not established. These genes do not appear in the list of hub gene drivers compiled by Xu et al.[26] for prostate adenocarcinoma. See Lam et al.[27] for a comprehensive listing of methylation-based biomarkers in prostate cancer.

2 Comparisons with Auto-weka predictors

In Supplementary Table S3 it is reported the performance of CVN versus the ML methods in the Auto-weka package (version 2.6)[28] for the Weka ML environment[29]. Following the same protocol in Pellegrini (2021)[30] the hyperparameters were optimized for Cohen kappa statistics over 27 base classification methods, 10 meta-methods, and two ensemble methods. Moreover, explicitly seven feature selection methods (including no selection) were applied. The reported kappa statistics are computed on the predictor trained on the train data and applied to the test data set. The overall outlook of this experiment with prostate cancer data is quite similar to that on breast cancer data reported in Pellegrini (2021)[30]. Over seven data sets corresponding to the seven selected fingerprints, CVN leads in four cases, ties in one, and loses in two. In each case a different Auto-weka algorithm is attaining the top Auto-weka performance, thus making it hard to pinpoint a single winner algorithm in the Auto-weka suite. Keeping experimental differences in mind, I can confirm the conclusion[30] that CVN has a level of performance at least comparable with existing ML methods. Moreover, as previously noted, CVN is a single easy-to-explain method that allows for a more uniform approach to the PRC prognosis problem over a wide spectrum of clinical conditions.

3 Performance on clinical stratifications

Rodriguez et al.[31] survey over 20 pre-treatment predictive models using various combinations of the three classical prognostic factors (PSA level, tumor stage, and Gleason Score). I have selected two of these stratification methods: one due to D'Amico et al.[32] and the NICE criterion[33] (National Institute for Health and Clinical Excellence) [NICE is an executive non-departmental public body of the Department of Health and Social Care in England that publishes guidelines in several areas.]. The two methods differ essentially in the thresholds for discriminating the Intermediate Risk (IR) class from the High Risk class (HR). As almost all patients from the TCGA cohort are at high risk according to both stratification criteria, only results on the independent cohorts with high OR and/or high value of kappa are reported (see Supplementary Table S4). The statistical significance is almost always attained, except for 3 cases due to the small number of patients involved. Overall CVN-based predictors can stratify well by year the HR patients in both systems. The performance of the CVN-based predictors on the NICE IR class is acceptable, but in general lower than for the HR class.

4 Random fingerprints

Several authors have noticed that fingerprints obtained by sampling uniformly at random in a pool of genes can have statically significant prognostic performance and sometimes outperform fingerprints obtained with other more elaborated (deterministic, or randomized) methodologies[34,35]. The methodology proposed in these studies is oblivious to the model-selection phase used in the determination of the competing multi-gene fingerprints. Here instead I apply a novel comparison methodology against randomly generated fingerprints that is sensitive to the model-selection phase so that random fingerprints and competing fingerprints are treated evenly. In Supplementary Table S5 it is reported the performance of the selected random fingerprint out of 100 randomly generated gene fingerprints, using the same model selection methodology used to attain the fingerprints listed in Supplementary Table S2 involving both Pareto-based and Ng-based model selection. In two cases (fp14, fp37) the random analog does not attain statistical significance either in OR or AUC p-values. In one case (fp0) the random analog attains statistical significance but has quite low performance. In four cases(fp1, fp20, fp30, and fp12) the random analogs are statistically significant and attain good performance, in terms of AUC and kappa measures, although they lag for the corresponding OR measures.

5 Overlap with published multi-gene signatures

In Supplementary Table S6 37 published multi-gene fingerprints developed for prostate cancer (for uses ranging from prognostic to predictive) are listed and compared for overlaps with the seven selected signatures. For fingerprint fp37 the genes associated with (closest to) the methylation sites are used. The published fingerprints have been selected using a comprehensive listing by Manjang et al.[36] by retaining fingerprints of size comparable with ours (i.e. < 100 genes), that are specific for prostate cancer. Moreover, fingerprints associated with commercial prognostic/predictive kits were added. The overlaps for most of the published fingerprints are minimal: gene CDK1 is shared with 3 published fingerprints and gene CHST1 with one. Interestingly, three genes (RB1, CDKN1B, MYC) are overlapping with the 27-genes fingerprint used by Gerhauser et al.[37] to identify early onset prostate cancer.

6 Independent validation cohorts: tumor-based samples

This section lists gives a high level description of the independent cohorts used in this study with tumor-based samples. Some technical details of the data acquisition technology are summarized in Supplementary Table S7.

MSKCC The data set MSKCC (Cancer Cell 2010) has been downloaded from cbioportal. Study data is also deposited in NCBI GEO under accession number GSE21032. Details of the patient selection and data processing are described by Taylor et al.[71]. In summary, a total of 218 tumor samples and 149 matched normal samples were obtained from patients treated with radical prostatectomy at Memorial Sloan-Kettering Cancer Center. All patients provided informed consent and samples were procured and the study was conducted under Memorial Sloan-Kettering Cancer Center Institutional Review Board approval. Clinical and pathologic data were entered and maintained in MSKCC prospective prostate cancer database. After radical prostatectomy, patients were followed with history, physical exam, and serum PSA testing every 3 months for the first year, every 6 months for the second year, and annually thereafter. Biochemical recurrence (BCR) was defined as PSA ≥ 0.2 ng/ml on two occasions. Note that in this study only the primary tumor-tissue data and clinical data are used.

GSE70769 Data from the study of Ross-Adams et al.[72] was obtained from NCBI GEO under accession number GSE70769. Briefly, the discovery cohort comprises 358 fresh frozen samples from 156 men, including 125 primary prostate cancer from

radical prostatectomy with matched benign tissue, 64 matched germline genomic DNA, 19 castrate-resistant prostate cancer (CRPC) from channel transurethral resection of the prostate, 13 with matched germline gDNA, and 12 independent benign samples from holmium laser enucleation of the prostate. Samples were prepared as described in Warren et al.[73]. Relative proportions of benign, epithelial, stromal, and tumor cells were determined by consultant histopathologists; samples with $\geq 20\%$ tumor and matched non-tumor cores (when available) were included. In this study, only data from the 125 primary prostate cancer, the 19 castrate-resistant prostate cancer (CRPC) cases, and clinical data are used.

GSE54460 Data from the study of Long et al.[70] was obtained from NCBI GEO under accession number GSE54460. See Long et al.[70] for more details on the patient selection process and the data processing techniques. In brief, this data set comprises RNA samples passing QC analysis from the Atlanta VA Medical Center (AVAMC), the U. Toronto Sunnybrook Research Centre (UT), and the Moffitt Cancer Center (MCC) in Tampa, FL. MCC Prostate cancer cases were men 21 years and older who had surgery (radical prostatectomy) between 1987 and 2003 for their disease at the MCC and had pathologically confirmed primary prostate cancer. AVAMC cases were patients with prostate cancer who underwent radical prostatectomy between 1990 and 2000. University of Toronto (UT) cases were patients with prostate cancer who underwent radical prostatectomy at the Sunnybrook Health Science Center between 1998 and 2006. These patients did not receive neoadjuvant or concomitant hormonal therapy before radical prostatectomy.

GSE46602 Data from the study of Mortensen et al.[69] was obtained from NCBI GEO under accession number GSE46602. Samples for this study were provided by the Aarhus prostate cancer project consisting of all patients undergoing radical prostatectomy at the Dept. of Urology, Aarhus University Hospital from 1995 to 2015. Clinical data were collected prospectively and recurrence status for all patients was updated before inclusion in the study[69]. The prostatectomy specimens were examined by a trained pathologist, the pathological stage was assessed and the Gleason grade of the tumor was determined. Serum PSA was measured prior to surgery by automated immunoassay using DPC Total PSA Immulite and expressed in ng/ml. Follow-up after surgery has been conducted by PSA measurements at 3, 6, and 12 months postoperatively and thereafter biannually. Subsequent biochemical failure was defined as a PSA ≥ 0.2 ng/ml. Biopsies were taken from the surgical specimen and immediately snap-frozen. Normal tissue samples were obtained from a different cohort of patients undergoing cystectomy. Note that in this study only the primary tumor-tissue data and clinical data are used.

GSE84042 Data from the study of Frazer et al.[74] was obtained from NCBI GEO under accession GSE84042 (including both methylation and mRNA gene expression data). More details on patient selection and data processing are in Frazer et al.[74]. Briefly, all patients in this cohort underwent either image-guided radiotherapy (IGRT) or radical prostatectomy (RadP), with curative intent for pathologically confirmed prostate cancer and were hormone naive at the time of definitive local therapy. In the IGRT sub-cohort, a single ultrasound-guided needle biopsy was obtained before the start of therapy, All fresh-frozen RadP specimens were obtained from the University Health Network (UHN) Pathology BioBank or the Genito-Urinary BioBank of the Centre Hospitalier Universitaire de Québec (CHUQ). All patients were of type N0M0 as an entry criterion for this cohort. For IGRT patients, BCR was defined as a rise in PSA concentration of more than 2.0 ng/ml above the nadir (after radiotherapy, PSA levels drop and stabilize at the nadir). For RadP patients, BCR was defined as two consecutive post-RadP PSA measurements of more than 0.2 ng/ml (backdated to the date of the first increase). If a patient has successful salvage radiation therapy, this is not BCR. If PSA continues to rise after radiation therapy, BCR is backdated to the time of the first PSA > 0.2. If the patient gets other salvage treatment (such as hormones or chemotherapy), this is considered BCR.

7 Independent validation cohorts: blood-based samples

This section lists gives a high level description of the independent cohorts used in this study with blood-based samples. Some technical details of the data acquisition technology are summarized in Supplementary Table S7.

GSE53922 PBMC or plasma samples were obtained from 117 patients with metastatic CRPC who were positive for human leukocyte antigen (HLA)-A2, A24, A3 supertype (A3, A11, A30, A31, and A33), or A26 and enrolled in clinical trials between February 2001 and April 2008 at the participating hospitals in Japan. Whole-genome gene expression profiles of peripheral blood mononuclear cells (PBMCs) in castration-resistant prostate cancer (CRPC) patients were measured before administration of Personalized peptide vaccination. More details on patient selection and data processing are in the study by Araki et al.[75]. Data were obtained from NCBI GEO under accession GSE53922.

GSE37199 Data from the study of Olmos et al.[76] was retrieved from NCBI GEO under accession number GSE37199.

Briefly, whole blood RNA samples were acquired from patients treated at The Royal Marsden Hospital NHS Foundation Trust (Sutton, UK) and The Beatson West of Scotland Cancer Centre (Glasgow, UK) between August 2007 and April

2008. Patients were enrolled in two groups: patients with advanced castration-resistant prostate (ACRPC) cancer; and (2) patients undergoing active surveillance in a prospective research trial (AS). All patients had a histological diagnosis of prostate cancer and provided informed and written consent for these studies, before sample collection. For each patient, 2·5 mL of peripheral venous blood was collected in 5 mL PAXgene tubes. All samples were taken at least 1 month after cessation of any prostate cancer therapy. Additionally, blood was collected 1 month after the first sampling in patients who had not yet been started on a new prostate-cancer treatment.

Whole-blood RNA was isolated and purified with the PAXgene Blood RNA Kit according to the manufacturer's instructions. RNA quality and quantity measures were done with a 2100 Bioanalyzer (Agilent Technologies, Palo Alto, CA, USA) and an ND-1000 spectrophotometer (Thermo Scientific, Newark, DE, USA), respectively.

As I could not access detailed follow-up data, I take the classification into AS and ACRPC patients as a proxy for the ground truth classification in low-risk high-risk sub-classes for PFS at 2 years. This choice is consistent with the value of the median OS from patients in clusters LPD1 and LPD2, which are rich in ACRPC patients with survival below 25 months[76].

8 CVN as an explanation tool

The process of building and selecting a CVN suitable for prognostic purposes is quite complex (the main CVN construction method is shown schematically in Supplementary Figure S1, and the optimization via the train-validation-testing pipeline and the leave-one-out plus bootstrapping pipeline is shown in Supplementary Figure S2). However, the predictor CVN obtained at the end is a simple object, just a collection of communities, where each community has a set of patients (from the training set) and each patient is originally labeled either high-risk or low-risk. Moreover, each community is associated with some features from the fingerprint, each with an interval of values (or a single value for discrete measures). We call this section of a community its *fingerprint profile*. Finally, a CVN has also a preferred voting scheme (the voting scheme giving the highest performance during the CVN construction and selection). When a new patient p is presented, its fingerprint value vector $f(p)$ must be measured. Next $f(p)$ is matched with the *fingerprint profile* of each community C. If the *fingerprint profile* of C matches the fingerprint vector $f(p)$ for p (i.e. the components of the vectors $f(p)$ fall within the corresponding intervals in the *fingerprint profile*), then the community C is allowed to vote with the preferred voting scheme. Note that the vote itself and its result does not depend on p, it is fixed for C, if selected. Finally, a majority vote is taken among all results collected from communities passing the matching phase. Supplementary Figure S3 gives a graphical example of the matching and voting procedure. Note that for patient p it is immediate to trace back the final prediction to the patients in the training set influencing the prediction. The communities in a CVN can be seen as a way to organize the training data as 'case studies' supporting the reasoning leading to a certain decision. This sort of explanation by example has precedent in how humans sometimes justify actions by analogy[77]. Using the taxonomies described in a recent survey[78] on explainable ML, CVN provides a type of 'rule-based' explanation for the prediction.

Babic et al.[79] warn of the danger of *ersatz understanding*, that is a rationale for a black-box prediction, which is not necessarily the actual reason behind that prediction or related causally to it. In our case the explanation provided by CVN is closely related to the way CVN concretely produces the outcome, thus CVN avoids the danger of giving spurious explanations.

In a scenario in which the recipient of the explanation is the medical personnel tasked with evaluating the prognostic evidence and the full clinical records for the training set of patients are available for inspection, a 'personalized' case report can be assembled in which only the training cases impacting the outcome are collected, displayed, and summarized. Eventual outliers biasing the result may be identified by human experts in this phase and removed from the voting process, thus creating a synergy between human expertise and CVN-based prediction.

9 Comparing the Pareto-based and the Ng-based model selection

In Supplementary Table S8, it is reported the comparative evaluation of the Pareto-based model selection vs. the Ng-based model selection. For the fingerprint fp30, both methods yield the same configuration, thus strongly supporting each other. For two fingerprints (fp12 and fp14) the Pareto solution is leading in all three performance measures (OR, kappa, and AUC). For two fingerprints (fp1 and fp20), the Ng solution is leading in all three performance measures. Finally, for three fingerprints (fp0, fp37, and fp160) the Pareto solution is leading in two performance measures out of three. As neither method dominates the other in all cases, both should be used when searching for the best model.

References

1. Liu, J. *et al.* An integrated tcga pan-cancer clinical data resource to drive high-quality survival outcome analytics. *Cell* **173**, 400–416 (2018).

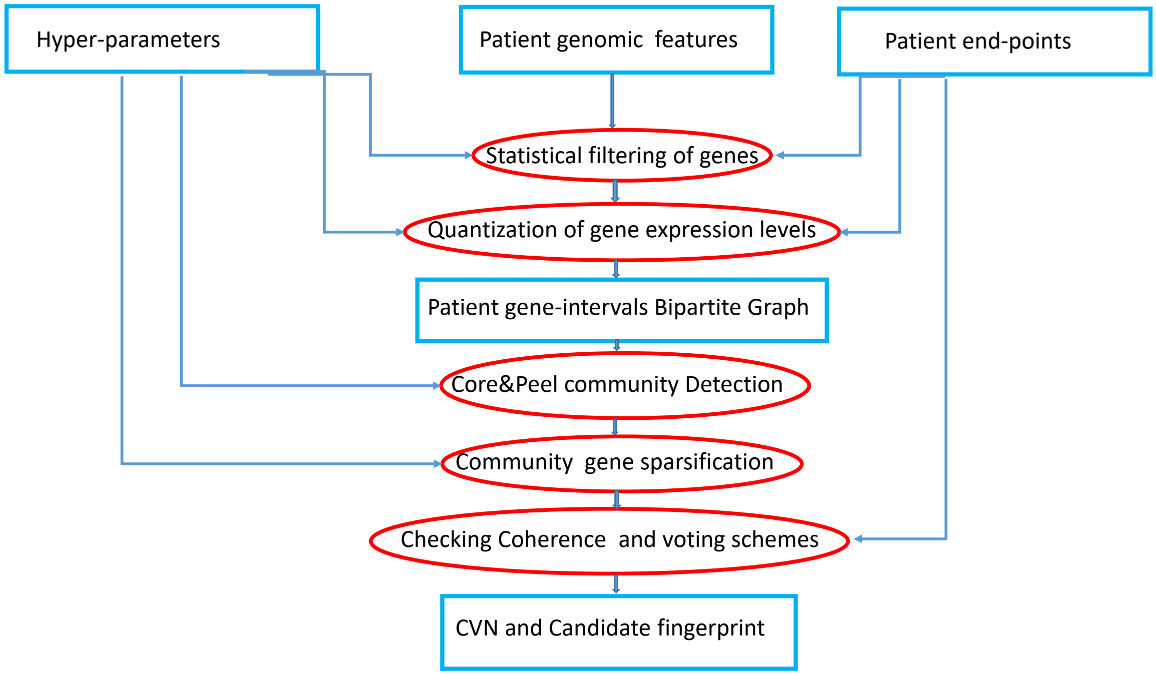

Figure S1. Schematic control flow for the CVN building and training algorithm. This pipeline is executed with different hyperparameters vectors so so produce a pool of candidate fingerprints corresponding to CVN with high coherence. Note that use of the patient end-points is limited to the initial statistical filtering of genes, for the gene expression quantization phase and for the final evaluation of coherence, but not for community detection and sparsification. Core&Peel is a graph community detection algorithm developed by Pellegrini et al.[80] here adapted to work on bipartite graphs.

2. Abbott, K. L. *et al.* The Candidate Cancer Gene Database: a database of cancer driver genes from forward genetic screens in mice. *Nucleic acids research* **43**, D844–8, DOI: 10.1093/nar/gku770 (2015).
3. Chu, J., Li, N. & Gai, W. Identification of genes that predict the biochemical recurrence of prostate cancer. *Oncol. Lett.* **16**, 3447–3452 (2018).
4. Ye, H. *et al.* Recognition of invasive prostate cancer using a ghrl polypeptide probe targeting ghsr in a mouse model in vivo. *Curr. Pharm. Des.* **26**, 1614–1621 (2020).
5. Wang, L.-Y. & Kung, H.-J. Male germ cell-associated kinase is overexpressed in prostate cancer cells and causes mitotic defects via deregulation of apc/ccdh1. *Oncogene* **31**, 2907–2918 (2012).
6. Orellana-Serradell, O., Herrera, D., Castellon, E. A. & Contreras, H. R. The transcription factor zeb1 promotes an aggressive phenotype in prostate cancer cell lines. *Asian J. Androl.* **20**, 294 (2018).
7. He, Y. *et al.* High rab11-fip4 expression predicts poor prognosis and exhibits tumor promotion in pancreatic cancer. *Int. journal oncology* **50**, 396–404 (2017).
8. Ye, C. *et al.* Lncrna eif3j-as1 functions as an oncogene by regulating mafg to promote prostate cancer progression. *J. Cancer* **13**, 146 (2022).
9. Wang, X.-L. *et al.* Knockdown of trim65 inhibits lung cancer cell proliferation, migration and invasion: A therapeutic target in human lung cancer. *Oncotarget* **7**, 81527 (2016).
10. Sirma, H. *et al.* Loss of cdkn1b/p27kip1 expression is associated with erg fusion-negative prostate cancer, but is unrelated to patient prognosis. *Oncol. letters* **6**, 1245–1252 (2013).
11. Xu, R. & Hu, J. The role of jnk in prostate cancer progression and therapeutic strategies. *Biomed. & Pharmacother.* **121**, 109679 (2020).
12. Koh, C. M. *et al.* Myc and prostate cancer. *Genes & cancer* **1**, 617–628 (2010).

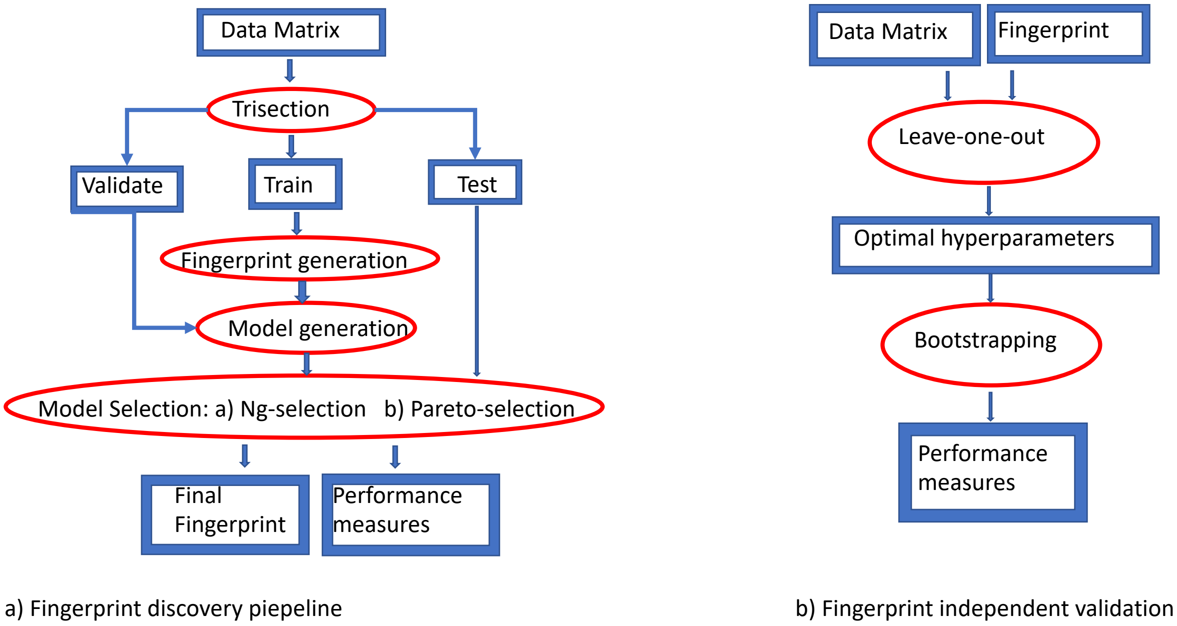

Figure S2. Schematic depiction of the two main software pipelines used in this work. a) Pipeline for fingerprint discovery and evaluation based on a train-validation-test scheme. b) Pipeline for evaluation of a given fingerprint on independent cohorts based on a leave-one-out and bootstrapping scheme.

13. Sharma, A. *et al.* The prostate metastasis suppressor gene ndrg1 differentially regulates cell motility and invasion. *Mol. oncology* **11**, 655–669 (2017).

14. Petrilli, A. M. & Fernández-Valle, C. Role of merlin/nf2 inactivation in tumor biology. *Oncogene* **35**, 537–548 (2016).

15. Han, W. *et al.* Rb1 loss in castration-resistant prostate cancer confers vulnerability to lsd1 inhibition. *Oncogene* **41**, 852–864 (2022).

16. Fritz, V. *et al.* Abrogation of de novo lipogenesis by stearoyl-coa desaturase 1 inhibition interferes with oncogenic signaling and blocks prostate cancer progression in micelipid synthesis and cancer. *Mol. cancer therapeutics* **9**, 1740–1754 (2010).

17. Qiu, L.-X. *et al.* The e-cadherin (cdh1)- 160 c/a polymorphism and prostate cancer risk: a meta-analysis. *Eur. J. Hum. Genet.* **17**, 244–249 (2009).

18. Kim, J. Y. *et al.* Nuclear interaction of smac/diablo with survivin at g2/m arrest prompts docetaxel-induced apoptosis in du145 prostate cancer cells. *Biochem. biophysical research communications* **350**, 949–954 (2006).

19. Nastaly, P. *et al.* Egfr as a stable marker of prostate cancer dissemination to bones. *Br. journal cancer* **123**, 1767–1774 (2020).

20. Tanaka, Y., Gavrielides, M. V., Mitsuuchi, Y., Fujii, T. & Kazanietz, M. G. Protein kinase c promotes apoptosis in lncap prostate cancer cells through activation of p38 mapk and inhibition of the akt survival pathway. *J. Biol. Chem.* **278**, 33753–33762 (2003).

21. Hussain, S. S. *et al.* Suppression of ribosomal protein rps6kb1 by nexrutine increases sensitivity of prostate tumors to radiation. *Cancer Lett.* **433**, 232–241 (2018).

22. Qiao, X.-R., Zhang, X., Mu, L., Tian, J. & Du, Y. Grb2-associated binding protein 2 regulates multiple pathways associated with the development of prostate cancer. *Oncol. letters* **20**, 1–1 (2020).

23. Chen, S., Xu, Y., Yuan, X., Bubley, G. J. & Balk, S. P. Androgen receptor phosphorylation and stabilization in prostate cancer by cyclin-dependent kinase 1. *Proc. Natl. Acad. Sci.* **103**, 15969–15974 (2006).

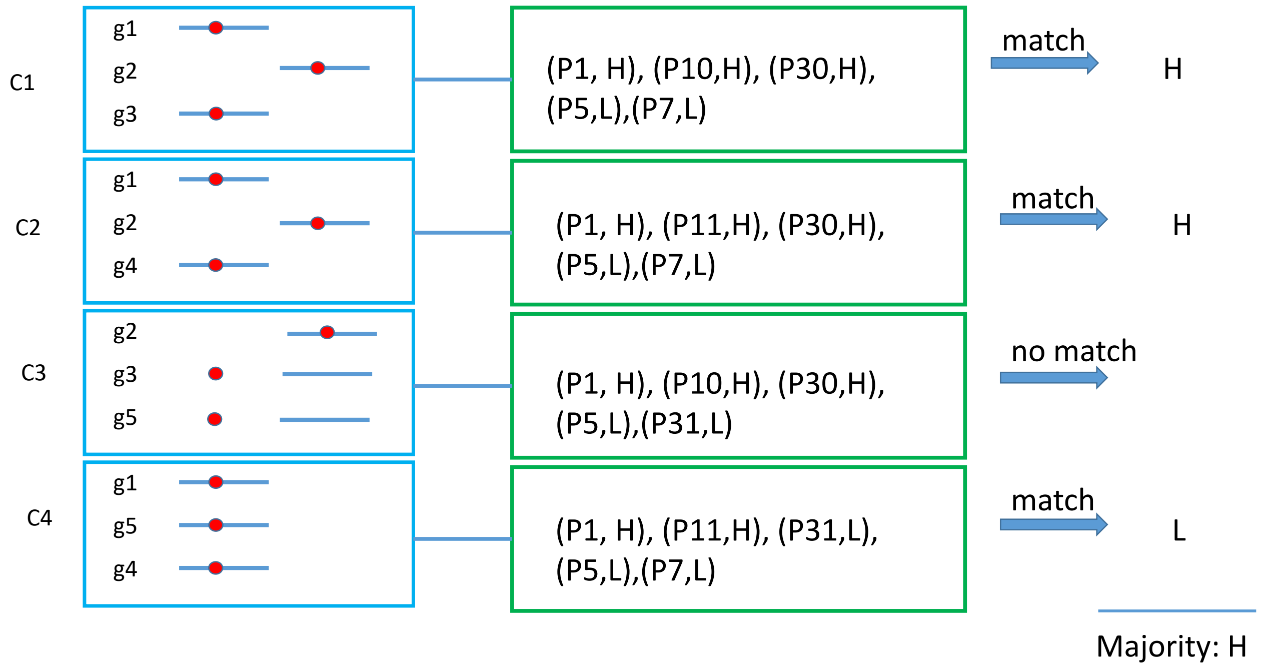

Figure S3. Example of a predictor composed on 4 communities for a fingerprint $f = (g1, g2, g3, g4, g5)$ of 5 genes. Training patients are indicated by the prefix P. H stands for high-risk of the event; L stands for low-risk of the event. Note that the fingerprint-profile of any community need not contain all 5 genes, but by construction it must contain at least 3 genes. Note also that that same training patient (with the same label) may appear multiple times in different communities. The components of the patient's fingerprint value vector $f(p)$ are denoted with the red dots. The preferred voting scheme for the communities in this example is majority. The final outcome is the majority of the collected community results.

24. Zheng, J.-Y. *et al.* Regulation of the expression of the prostate-specific antigen by claudin-7. *The J. membrane biology* **194**, 187–197 (2003).

25. Shi, X.-B. *et al.* An androgen-regulated mirna suppresses bak1 expression and induces androgen-independent growth of prostate cancer cells. *Proc. Natl. Acad. Sci.* **104**, 19983–19988 (2007).

26. Xu, N. *et al.* Identification of key dna methylation-driven genes in prostate adenocarcinoma: an integrative analysis of tcga methylation data. *J. translational medicine* **17**, 1–15 (2019).

27. Lam, D., Clark, S., Stirzaker, C. & Pidsley, R. Advances in prognostic methylation biomarkers for prostate cancer. *Cancers* **12**, 2993 (2020).

28. Kotthoff, L., Thornton, C., Hoos, H. H., Hutter, F. & Leyton-Brown, K. Auto-weka 2.0: Automatic model selection and hyperparameter optimization in weka. *J. Mach. Learn. Res.* **18**, 826–830 (2017).

29. Frank, E. *et al.* Weka-a machine learning workbench for data mining. In *Data mining and knowledge discovery handbook*, 1269–1277 (Springer, 2009).

30. Pellegrini, M. Accurate prediction of breast cancer survival through coherent voting networks with gene expression profiling. *Sci. Reports* **11**, 1–15 (2021).

31. Rodrigues, G. *et al.* Pre-treatment risk stratification of prostate cancer patients: A critical review. *Can. Urol. Assoc. J.* **6**, 121 (2012).

32. D'Amico, A. V. *et al.* Biochemical outcome after radical prostatectomy, external beam radiation therapy, or interstitial radiation therapy for clinically localized prostate cancer. *Jama* **280**, 969–974 (1998).

33. Graham, J., Baker, M., Macbeth, F. & Titshall, V. Diagnosis and treatment of prostate cancer: summary of nice guidance. *Bmj* **336**, 610–612 (2008).

34. Venet, D., Dumont, J. E. & Detours, V. Most random gene expression signatures are significantly associated with breast cancer outcome. *PLoS computational biology* **7**, e1002240 (2011).

35. Manjang, K. *et al.* Prognostic gene expression signatures of breast cancer are lacking a sensible biological meaning. *Sci. reports* **11**, 1–18 (2021).

36. Manjang, K., Yli-Harja, O., Dehmer, M. & Emmert-Streib, F. Limitations of explainability for established prognostic biomarkers of prostate cancer. *Front. Genet.* **12** (2021).

37. Gerhauser, C. *et al.* Molecular evolution of early-onset prostate cancer identifies molecular risk markers and clinical trajectories. *Cancer Cell* **34**, 996–1011 (2018).

38. Agell, L. *et al.* A 12-gene expression signature is associated with aggressive histological in prostate cancer: Sec14l1 and tceb1 genes are potential markers of progression. *The Am. journal pathology* **181**, 1585–1594 (2012).

39. Bibikova, M. *et al.* Expression signatures that correlated with gleason score and relapse in prostate cancer. *Genomics* **89**, 666–672 (2007).

40. Bismar, T. A. *et al.* Defining aggressive prostate cancer using a 12-gene model. *Neoplasia* **8**, 59–68 (2006).

41. Chen, X. *et al.* Comprehensive analysis of biomarkers for prostate cancer based on weighted gene co-expression network analysis. *Medicine* **99** (2020).

42. Chen, X. *et al.* An accurate prostate cancer prognosticator using a seven-gene signature plus gleason score and taking cell type heterogeneity into account. *PLOS ONE* **7**, 1–7, DOI: 10.1371/journal.pone.0045178 (2012).

43. Cheville, J. C. *et al.* Gene panel model predictive of outcome in men at high-risk of systemic progression and death from prostate cancer after radical retropubic prostatectomy. *J. Clin. Oncol.* **26**, 3930 (2008).

44. Cuzick, J. *et al.* Prognostic value of an rna expression signature derived from cell cycle proliferation genes in patients with prostate cancer: a retrospective study. *The lancet oncology* **12**, 245–255 (2011).

45. Glinsky, G. V., Berezovska, O., Glinskii, A. B. *et al.* Microarray analysis identifies a death-from-cancer signature predicting therapy failure in patients with multiple types of cancer. *The J. clinical investigation* **115**, 1503–1521 (2005).

46. Irshad, S. *et al.* A molecular signature predictive of indolent prostate cancer. *Sci. translational medicine* **5**, 202ra122–202ra122 (2013).

47. Larkin, S. *et al.* Identification of markers of prostate cancer progression using candidate gene expression. *Br. journal cancer* **106**, 157–165 (2012).

48. Li, F., Ji, J.-P., Xu, Y. & Liu, R.-L. Identification a novel set of 6 differential expressed genes in prostate cancer that can potentially predict biochemical recurrence after curative surgery. *Clin. Transl. Oncol.* **21**, 1067–1075 (2019).

49. Long, Q. *et al.* Protein-coding and microrna biomarkers of recurrence of prostate cancer following radical prostatectomy. *The Am. journal pathology* **179**, 46–54 (2011).

50. Nakagawa, T. *et al.* A tissue biomarker panel predicting systemic progression after psa recurrence post-definitive prostate cancer therapy. *PloS one* **3**, e2318 (2008).

51. Ramaswamy, S., Ross, K. N., Lander, E. S. & Golub, T. R. A molecular signature of metastasis in primary solid tumors. *Nat. genetics* **33**, 49–54 (2003).

52. Reddy, G. K. & Balk, S. P. Clinical utility of microarray-derived genetic signatures in predicting outcomes in prostate cancer. *Clin. Genitourin. Cancer* **5**, 187–189 (2006).

53. Ross, R. W. *et al.* A whole-blood rna transcript-based prognostic model in men with castration-resistant prostate cancer: a prospective study. *The lancet oncology* **13**, 1105–1113 (2012).

54. Sharma, N. L. *et al.* The androgen receptor induces a distinct transcriptional program in castration-resistant prostate cancer in man. *Cancer cell* **23**, 35–47 (2013).

55. Singh, D. *et al.* Gene expression correlates of clinical prostate cancer behavior. *Cancer cell* **1**, 203–209 (2002).

56. Song, Z. *et al.* The identification of potential biomarkers and biological pathways in prostate cancer. *J. Cancer* **10**, 1398 (2019).

57. Stephenson, A. J. *et al.* Integration of gene expression profiling and clinical variables to predict prostate carcinoma recurrence after radical prostatectomy. *Cancer: Interdiscip. Int. J. Am. Cancer Soc.* **104**, 290–298 (2005).

58. Talantov, D. *et al.* Gene based prediction of clinically localized prostate cancer progression after radical prostatectomy. *The J. urology* **184**, 1521–1528 (2010).

59. Wang, L.-Y. *et al.* Biomarkers identified for prostate cancer patients through genome-scale screening. *Oncotarget* **8**, 92055 (2017).

60. Wu, C.-L. *et al.* Development and validation of a 32-gene prognostic index for prostate cancer progression. *Proc. Natl. Acad. Sci.* **110**, 6121–6126 (2013).

61. Yu, J. *et al.* A polycomb repression signature in metastatic prostate cancer predicts cancer outcome. *Cancer research* **67**, 10657–10663 (2007).

62. Knezevic, D. *et al.* Analytical validation of the oncotype dx prostate cancer assay–a clinical rt-pcr assay optimized for prostate needle biopsies. *BMC genomics* **14**, 1–12 (2013).

63. Erho, N. *et al.* Discovery and validation of a prostate cancer genomic classifier that predicts early metastasis following radical prostatectomy. *PloS one* **8**, e66855 (2013).

64. Zhao, S. G. *et al.* Development and validation of a 24-gene predictor of response to postoperative radiotherapy in prostate cancer: a matched, retrospective analysis. *The lancet oncology* **17**, 1612–1620 (2016).

65. Shipitsin, M. *et al.* Identification of proteomic biomarkers predicting prostate cancer aggressiveness and lethality despite biopsy-sampling error. *Br. journal cancer* **111**, 1201–1212 (2014).

66. Goh, L. K. *et al.* Diagnostic and prognostic utility of a dna hypermethylated gene signature in prostate cancer. *PLoS One* **9**, e91666 (2014).

67. Mundbjerg, K. *et al.* Identifying aggressive prostate cancer foci using a dna methylation classifier. *Genome biology* **18**, 1–15 (2017).

68. Jeyapala, R. *et al.* An integrative dna methylation model for improved prognostication of postsurgery recurrence and therapy in prostate cancer patients. In *Urologic Oncology: Seminars and Original Investigations*, vol. 38, 39–e1 (Elsevier, 2020).

69. Mortensen, M. M. *et al.* Expression profiling of prostate cancer tissue delineates genes associated with recurrence after prostatectomy. *Sci. reports* **5**, 1–11 (2015).

70. Long, Q. *et al.* Global transcriptome analysis of formalin-fixed prostate cancer specimens identifies biomarkers of disease recurrence. *Cancer research* **74**, 3228–3237 (2014).

71. Taylor, B. S. *et al.* Integrative genomic profiling of human prostate cancer. *Cancer cell* **18**, 11–22 (2010).

72. Ross-Adams, H. *et al.* Integration of copy number and transcriptomics provides risk stratification in prostate cancer: a discovery and validation cohort study. *EBioMedicine* **2**, 1133–1144 (2015).

73. Warren, A. Y. *et al.* Method for sampling tissue for research which preserves pathological data in radical prostatectomy. *The Prostate* **73**, 194–202 (2013).

74. Fraser, M. *et al.* Genomic hallmarks of localized, non-indolent prostate cancer. *Nature* **541**, 359–364 (2017).

75. Araki, H. *et al.* Haptoglobin promoter polymorphism rs5472 as a prognostic biomarker for peptide vaccine efficacy in castration-resistant prostate cancer patients. *Cancer Immunol. Immunother.* **64**, 1565–1573 (2015).

76. Olmos, D. *et al.* Prognostic value of blood mrna expression signatures in castration-resistant prostate cancer: a prospective, two-stage study. *The lancet oncology* **13**, 1114–1124 (2012).

77. Zachary, C. L. The mythos of model interpretability. *Commun. ACM* 1–6 (2016).

78. Guidotti, R. *et al.* A survey of methods for explaining black box models. *ACM computing surveys (CSUR)* **51**, 1–42 (2018).

79. Babic, B., Gerke, S., Evgeniou, T. & Cohen, I. G. Beware explanations from ai in health care. *Science* **373**, 284–286 (2021).

80. Pellegrini, M., Baglioni, M. & Geraci, F. Protein complex prediction for large protein protein interaction networks with the core&peel method. *BMC bioinformatics* **17**, 372 (2016).

fp	gene	Protein Atlas	note	COSMIC
fp0	CHST1	renal, liver		no
	GHRL	-		mouse gene
	MAK	-		no
	RAB11FIP4	renal,stomach,colorectal		no
	RPEL1	-		no
	ZEB1	renal		census tier 2
fp1	ASH1L-AS1	renal		(ASH1L) mouse gene
	PIFO	renal, pancreatic		mouse gene
	DBN1	renal		no
	HRSP12 (RIDA)	liver		mouse gene
	MAFG	liver, endometrial		no
	SNORA18		snRNA	
	TRIM65	liver, renal		mouse gene
fp14	CDH1	renal		hallmark
	DIABLO	renal		
	EGFR	urothelial		hallmark
	GAB2	renal		mouse gene
	PRKCA	-		mouse gene
	RPS6KB1	renal		mouse gene
fp12	CDKN1B	liver, colorectal,renal		hallmark
	MAPK9	colorectal		no
	MYC	renal,urothelial,ovarian		hallmark
	NDRG1	liver, renal		hallmark
	NF2	renal		hallmark
	RB1	ovarian		hallmark
	SCD	renal, urothelial		no
fp30	CDK1	renal,liver,pancreatic,lung, cervical		no
	CLDN7	renal, thyroid, stomach		mouse gene
fp20	BAK1	renal, endometrial, liver, lung		no
	PTCHD4	renal		no
	FANCC	renal		census tier 1
	FBRSL1	renal, urothelial, prostate		no
	OMP	-		no
	SULT1C3	-		no
fp37	CCR10	-		no
	NRN1	renal cancer		no
	NPR3	renal cancer		no
	C14orf23		LINC	
	ATXN7L1	-		no

Table S1. Cancer-related annotations for the genes in the pool of seven fingerprints selected by CVN. The table reports the fingerprint identifier (fp), the unique genes in the fingerprint, the cancer types for which the gene has prognostic power for Overall Survival, according to The Human Protein Atlas database - https://www.proteinatlas.org - (Protein Atlas), for non-coding genes the molecular type (note), the most stringent annotation of the gene in the COSMIC (Catalogue Of Somatic Mutations In Cancer) database - https://cancer.sanger.ac.uk/cosmic/ (COSMIC). Note that fp30 shares many genes with fp12, which are reported once.

Fp	Time	Data	genes	size
Fp0	2-3	mrna	CHST1 , GHRL , MAK , RAB11FIP4 , RPEL1 , ZEB1	6
Fp1	3-4	mrna	ASH1L-AS1 , C1orf88 , DBN1 , HRSP12 , MAFG , SNORA18 , TRIM65	7
Fp12	2-3	rppa	CDKN1B , MAPK9 , MYC , NDRG1 , NF2 , RB1 , SCD	7
Fp14	4-5	rppa	CDH1 , DIABLO , EGFR , GAB2 , PRKCA , RPS6KB1	6
Fp30	3-4	rppa	CDK1 , CDKN1B , CLDN7 , MYC , NF2 , SCD	6
Fp20	2-3	mrna+rppa	BAK1, PTCHD4, FANCC, FBRSL1, OMP, SULT1C3, CDKN1B	7
Fp37	3-4	methyl	cg02928644, cg03062002, cg11504897, cg11620238, cg22337128, cg22661239	6
Fp160	3-4	clinical+rppa	PSA, Tumor Stage, Gleason primary score, NF2, CDKN1B	5

Table S2. Listing of seven fingerprints with reference to time frame of high to low risk stratification in years, and to the omic data type. Genes are reported in HUGO nomenclature. Methylation loci are denoted with Illumina HumanMethylation450 BeadChip identification labels. rppa stands for Reverse Phase Protein Array.

file n.	n. pats	CVN kappa	autoweka kappa	algorithm	feature selection
0	53	0.29	**0.32**	Bagging	Corr-Ranker
1	37	**0.54**	0.45	SGD	Corr-Ranker
12	39	**0.47**	**0.47**	Random Tree	J48-Ranker
14	19	0.49	**0.57**	AdaBoost	Cfs-best
30	25	**0.53**	0.33	Simple Logistic	All genes
20	39	**0.43**	0.21	Lazy LWL	J48-Ranker
37	31	**0.59**	0.17	Lazy Ibk	Corr-ranker

Table S3. Comparative results of CVN and the Autoweka ML environment. The table reports the input file ID (file n.) corresponding to the seven fingerprints in Supplementary Table S2, the number of patients in the test set (n. pats), the value of Cohen's kappa for CVN and for Autoweka, along with the algorithm and the feature selection method attaining it. Autoweka is trained on the corresponding training set via ten-fold cross-validation.

fp	Dataset	Time	stratum	n. pats	n.p.	OR	P-val	kappa
fp37	GSE84042	4-5	nice IR	48	4	9.3	0.04	0.29
fp37	GSE84042	4-5	nice HR	41	9	27	0.0003	0.66
fp37	GSE84042	4-5	damico HR	79	11	20.5	4.00E-005	0.5
fp0	GSE46602	2-3	damico HR	28	1	8.12	0.07	0.31
fp0	GSE46602	2-3	nice HR	20	1	4.2	0.03	0.23
fp1	GSE46602	2-3	damico HR	28	0	36	0.0004	0.71
fp1	GSE46602	2-3	nice IR	9	0	7	0.37	0.6
fp1	GSE46602	2-3	nice HR	20	0	33	0.004	0.68
fp12	GSE46602	2-3	damico HR	28	2	66	0.0001	0.76
fp12	GSE46602	2-3	nice IR	9	2	5	0.46	0.58
fp12	GSE46602	2-3	nice HR	20	0	38.5	0.002	0.79
fp20	GSE46602	3-4	damico HR	28	0	20	0.003	0.61
fp20	GSE46602	3-4	nice HR	22	0	14	0.02	0.54
fp1	GSE46602	3-4	damico HR	28	0	28	0.001	0.6
fp1	GSE46602	3-4	nice HR	22	0	30	0.009	0.63
fp14	GSE46602	3-4	damico HR	28	2	17.3	0.003	0.6
fp14	GSE46602	3-4	nice HR	22	2	12	0.03	0.52
fp0	GSE70769	2-3	damico HR	17	2	26	0.06	1
fp0	GSE70769	2-3	nice IR	22	3	11	0.1	0.53
fp0	GSE70769	2-3	nice HR	16	2	13	0.24	1

Table S4. Performance of fingerprints and corresponding bootstrap consensus predictors on subsets of patients identified as High risk (HR) or Intermediate Risk (IR) by two stratification schemes based on tumor stage, PSA and Gleason score: the D'Amico scheme[32] and the NICE scheme[33]. The table reports the fingerprint identifier (Fp), the independent cohort identifier (Dataset), the time frame of high to low risk stratification in years for the CVN method (Time) the number of patients in the subset of patients (n. pats), the number of no predictions (n.p.), the odds ratio (OR), its p-value (P-val), and the Cohen's kappa (kappa) for the bootstrap consensus predictor on the subset of patients.

file n.	fp size	n. pats	n.p.	OR	P-val	kappa	AUC	AUC-pval	lookahead
0	6	53	0	4	0.08	0.23	0.65	0.04	0
1	7	37	0	11.3	0.003	0.47	0.79	0.001	3
12	7	39	0	9.33	0.04	0.4	0.83	0.004	2
14	6	19	0	1.5	1	0.09	0.65	0.14	0
30	6	25	4	14	0.05	0.48	0.74	0.04	2
20	7	39	2	8.66	0.02	0.41	0.7	0.03	2
37	6	31	4	0.3	0.6	-0.17	0.49	0.54	0

Table S5. Performance evaluation of randomly generated fingerprints. The random fingerprint size is fixed equal to the size of the corresponding fingerprint in Table S2. The random sampling is performed on the genes passing the initial statistical filter. The table reports the performance of the model with best OR among the Pareto-based and the Ng-based selected models. The table reports the input file ID (file n.) corresponding to the seven fingerprints in Table S2, the fixed size of the sampled fingerprints (fp size), the number of patients in the test set (n. pats), the number of no predictions (n.p.), the odds ratio (OR), its p-value (P-val), the Cohen's kappa (kappa), the area under the curve (AUC) value, its p-value (AUC-pval), and the lookahead number. The lookahead number is default 0 for models Ng-based.

N.	ID	size	ref.	overlap	kit
1	AGELL	12	38		
2	BIBIKOVA	16	39		
3	BISMAR	12	40		
4	CHEN	4	41		
5	CHEN-2	7	42		
6	CHEVILLE	2	43		
7	CHU	8	3	CHST1	Prolaris
8	CUZICK	31	44	CDK1	
9	GLINSKY	11	45		
10	IRSHAD	19	46		
11	IRSHAD-2	3	46		
12	LARKIN	7	47		
13	LI	6	48		
13	LONG	12	49		
15	NAKAGAWA	17	50		
16	RAMASWAMY	16	51		
17	REDDY	16	52		
18	ROSS	6	53		
19	SHARMA	15	54		
20	SINGH	5	55		
21	SONG	15	56	CDK1	
22	STEPHENSON	10	57		
23	TALANTOV	3	58		
24	WANG	43	59		
25	WU	29	60	CDK1	
26	YU	14	61		
27	KNEZEVIC	12	62		Oncotype
28	EHRO	19	63		Decipher
29	ZHAO	24	64		PORTOS
30	SHIPITSIN	12	65		ProMark
31	XU	20	26		
32	GERHAUSER	27	37	RB1, CDKN1B, MYC	
33	GOH	46	66	CDKN1B	PHYMA
34	Mundbjerg	18	67		
35	Jeyapala	4	68		
36	MORTENSEN	12	69		
37	LONG_2014	24	70		

Table S6. Composition comparison of the CVN fingerprints with published fingerprints in prostate cancer. The table lists progressive number (N.), the fingerprint ID by name of the first author (ID), the published fingerprint size (size), a bibliographical reference (ref.), the genes in common with any of the 7 selected fingerprints (overlap), and a note of eventual commercial name of an associated prognostic kit (kit). The intersection takes into account gene name aliases as reported by GeneCards - The Human Gene Database https://www.genecards.org/

ID	E.P.	Platform	n. pats
TCGA-PRAD	PFS	Illumina HiSeq 2000 (mRNA)	495
		Illumina HumanMethylation450 BeadChip (methyl)	
		Reverse Phase Protein Array (RPPA) Expression (proteomics)	
MSKCC	DFS	Affymetrix Human Exon 1.0 ST arrays	131
GSE70769	BCR	Illumina HumanHT-12 V4.0 expression beadchip	92
GSE54460	OS	Human 6k Transcriptionally Informative Gene Panel for DASL	106
GSE46602	BCR	Affymetrix Human Genome U133 Plus 2.0 Array	36
GSE53922	OS	Illumina HumanWG-6 v3.0 expression beadchip	112
GSE84042	BCR	Illumina HumanMethylation450 BeadChip	160
		Affymetrix Human Transcriptome Array 2.0	
		Affymetrix Human Gene 2.0 ST Array	
GSE37199	HR-LR	Affymetrix Human Genome U133 Plus 2.0 Array	107

Table S7. Technological platforms for measuring molecular species in the discovery cohort (TCGA-PRAD) and in the independent cohorts. The platforms corresponding to the molecular data (mRNA, rppa, methylation) used in this study are reported. The table lists the cohort identifier (ID), the end point event (E.P.), the technological platforms (Platform), and the raw number of patients of the cohort (n. pats). Number of patients refers to the raw initial number in the repository, before the application of data filters and restrictions.

		Pareto					Ng			
Fp	n. pats	n.a.	OR	kappa	AUC	lookahead	n.a.	OR	kappa	AUC
Fp_0_Pareto	53	**1**	**13**	0.29	**0.72**	2	14	5.6	**0.30**	0.71
Fp_1_Ng	37	**3**	3.25	0.28	0.63	7	**3**	**20**	**0.54**	**0.70**
Fp_12_Pareto	39	**3**	**21**	**0.47**	**0.62**	1	6	1.33	0.05	0.61
Fp_14_Pareto	19	**2**	**12**	**0.49**	**0.71**	1	5	4.0	0.36	0.68
Fp_30_Ng	25	**0**	**18.75**	**0.53**	**0.72**	1	**0**	**18.75**	**0.53**	**0.72**
Fp_20_Ng	39	**0**	10.0	0.28	0.64	4	2	**17.14**	**0.43**	**0.79**
Fp_37_Pareto	31	**0**	**16**	**0.59**	0.78	1	3	8.25	0.43	**0.84**
Fp_160_Pareto	25	**1**	**22.75**	**0.64**	0.870	1	3	18	0.60	**0.879**

Table S8. Comparative evaluation of the Ng and Pareto selection outcomes for the selected fingerprints. In Bold the best values. The table reports the values of the Odds Ratio (OR), Cohen's kappa (kappa), the ROC Area Under the Curve (AUC), the number of missing answers (n.a.), and the lookahead number for the Pareto selection. The results shown are for the TCGA-PRAD test data.

	Train		Validation		Testing	
PFS event						
Num	122	-	64	-	55	-
0:CENSORED	69	56%	45	70%	34	61%
1:PROGRESSION	53	43%	19	29%	21	38%
no data	0	-	0	-	0	-
Tumor stage						
Num	122	-	63	-	54	-
T34	86	70%	40	63%	38	70%
T2	36	29%	23	36%	16	29%
no data	0	-	1	-	1	-
Lymph node stage						
Num	106	-	57	-	47	-
N1	21	19%	12	21%	7	14%
N0	85	80%	45	78%	40	85%
no data	16	-	7	-	8	-
Radiation Therapy						
Num	119	-	62	-	54	-
No	100	84%	56	90%	48	88%
Yes	19	15%	6	9%	6	11%
no data	3	-	2	-	1	-
Gleason sum						
Num	81	-	49	-	36	-
LR	55	67%	34	69%	27	75%
HR	26	32%	15	30%	9	25%
no data	41	-	15	-	19	-

Table S9. Categorical attributes of the TCGA PRAD patients. Progression free survival (PFS) event. 1=Progression, 0=Censored. Tumor stage. T34 includes T3A, T3B, T3C and T4. T2 includes T2A, T2B and T2C. Lymph Node Stage (American Joint Committee on Cancer Code). Reviewed Gleason Sum, LR (Low Risk) corresponds to levels 6 and 7, HR (High Risk) corresponds to levels 8,9 and 10.

	Train	Validation	Testing
PSF (month)			
num	122	64	55
mean	44.01	48.35	47.09
std dev	26.04	26.05	32.89
median	44.97	45.32	44.88
min	1.68	3.22	2.60
max	122.17	141.20	165.17
Age (years)			
num	118	62	55
mean	61.97	62.48	60.47
std dev	6.36	5.98	6.32
median	63.00	63.00	61.00
min	45.00	51.00	47.00
max	79.00	77.00	73.00
TMB			
num	81	49	36
mean	1.00	1.00	1.02
std dev	1.47	1.36	1.00
median	0.70	0.73	0.78
min	0.03	0.00	0.20
max	11.83	8.47	5.97
Last follow-up (months)			
num	121	62	53
mean	133.26	137.15	142.74
std dev	57.06	60.78	71.64
median	121.00	126.00	124.00
min	0.00	29.00	24.00
max	309.00	357.00	418.00
PSA			
num	114	62	53
mean	0.69	0.90	1.12
std dev	2.58	2.46	5.43
median	0.10	0.10	0.10
min	0.00	0.00	0.00
max	19.80	12.01	39.80

Table S10. Numerical attributes of the TCGA PRAD patients. Progression free survival (PFS) time in months. Age at first diagnosis (years). Tumor mutation burden (TMB) nonsynonymous. Time interval from the date of initial pathologic diagnosis to the date of last followup (in months). Pre-operatory value of PSA.
